# Trastuzumab-emtansine versus other anti-HER2 regimens in early or unresectable or metastatic HER-2 positive breast cancer: systematic review and network meta-analysis

**DOI:** 10.17843/rpmesp.2024.411.13351

**Published:** 2024-03-27

**Authors:** Agustín Ciapponi, Ariel Bardach, Carla Colaci, Federico Rodríguez Cairoli, Fernando Argento, Ernesto Korbenfeld, Sebastián García Martí

**Affiliations:** 1 Instituto de Efectividad Clínica y Sanitaria, Buenos Aires, Argentina. Instituto de Efectividad Clínica y Sanitaria Buenos Aires Argentina; 2 Health Technology Assessment and Health Economics Department of the Institute for Clinical Effectiveness and Health Policy (IECS-CONICET), Buenos Aires, Argentina. Health Technology Assessment and Health Economics Department of the Institute for Clinical Effectiveness and Health Policy (IECS-CONICET) Buenos Aires Argentina; 3 Hospital Británico de Buenos Aires, Buenos Aires, Argentina. Hospital Británico de Buenos Aires Buenos Aires Argentina

**Keywords:** HER2 Genes, Breast Cancer, Network Meta-Analysis, Systematic Review, Trastuzumab Emtansine

## Abstract

**Objective.:**

We aimed to study the efficacy and safety of trastuzumab-emtansine (T-DM1) versus other anti-HER2 therapies in HER2+ breast cancer (BC).

**Materials and Methods.:**

We performed a network meta-analysis (NMA) of randomized controlled trials (RCTs). Our study focused on patients undergoing treatment for unresectable locally advanced breast cancer (LABC) or metastatic breast cancer (mBC), which included regimens involving trastuzumab and taxanes. Additionally, we considered cases within the first 6 months of treatment for HER2+ early breast cancer (EBC).

**Results.:**

A total of 23 RCTs and 41 reports were included in our analysis. LABC and mBC showed no statistically significant difference in any of the comparisons of T-DM1 versus the other anti-HER2+ therapies. When assessing progression-free survival (PFS), trastuzumab-deruxtecan (T-DXd) and PyroCap demonstrated greater efficacy compared to other treatments (Hazard Ratio [HR]: 3.57; 95% confidence interval [CI]: 2.75-4.63 and HR: 1.82; 95% CI: 1.35-2.44; respectively), while T-DM1 alone exhibited superior effectiveness compared to LapCap (HR: 0.65; 95% CI: 0.55-0.77), TrasCap (HR: 0.65; 95% CI: 0.46-0.91), LapCapCitu (HR: 0.60; 95% CI: 0.33-1.10), Nera (HR: 0.55; 95% CI: 0.39-0.77), and Cap (HR: 0.37; 95% CI: 0.28-0.49).

**Conclusions.:**

NMA allows a ranking based on the comparative efficacy and safety among the interventions available. Although superior to other schemes, T-DM1 showed a lower efficacy performance in PFS and overall response rate and a trend towards worse overall survival than T-DXd.

## INTRODUCTION

Worldwide, breast cancer (BC) is the most common cancer and the leading cause of cancer-related death in women. Each year, more than two million cases are diagnosed and it is responsible for more than 600 000 deaths worldwide. Approximately half of the cases and 60% of the deaths occur in developing countries ^1^.

Selection of the type, schedule and sequence of treatment depends on the extent of disease and various clinical factors, with overexpression of human epidermal growth factor receptor 2 (HER2+) being a crucial consideration ^2^. HER2+ BC is characterised by aggressive behaviour, which results in shorter disease-free and overall survival (OS) in both early and advanced stages. The approval of trastuzumab (Herceptin®) in 1998 changed the treatment paradigm, emphasising prolongation of progression-free survival (PFS) ^3^^-^^5^ and OS ^6^^-^^8^. In 2006, the US Food and Drug Administration (FDA) approval expanded the use of trastuzumab in early-stage disease based on demonstrated benefits for disease-free survival (DFS) and OS in large phase III trials ^7^^,^^9^^,^^10^.

In recent years, new anti-HER2 drugs with diverse mechanisms have been introduced, including monoclonal antibodies, tyrosine kinase inhibitors and conjugated monoclonal antibodies such as trastuzumab-emtansine (T-DM1) and trastuzumab-deruxtecan (T-DXd). T-DM1 (Ka-dcyla®), the first monoclonal antibody conjugate, gained regulatory approval in 2013 for locoregionally advanced metastatic or unresectable breast cancer. Its approval by the FDA (November 2013) and the European Medicines Agency (EMA) marked a significant advance in the treatment of advanced HER2+ BC based on the results of the pivotal EMILIA study ^11^. In May 2019, the FDA approved T-DM1 as adjuvant treatment of early HER2+ disease in patients with persistent invasive residual disease after trastuzumab- and taxane-based neoadjuvant therapy, as demonstrated in the KATHERINE trial ^12^.

Recent research has explored anti-HER2 therapies, expanding the indications of T-DM1 for advanced HER2+ disease (previously treated and progressed with trastuzumab and taxanes). These include monoclonal antibodies directed against the extracellular domain of HER2 ^6^^-^^8^^,^^13^^,^^14^, small-molecule inhibitors of HER family receptor tyrosine kinase activity ^15^^-^^19^ and recently approved conjugated monoclonal antibodies such as T-DXd ^20^. Studies have also explored combinations with chemotherapeutics such as capecitabine ^21^ and vinorelbine ^22^, as well as specific target drugs such as everolimus (mTOR inhibitor) ^23^. Previous systematic reviews and meta-analyses have evaluated the efficacy and safety of T-DM1 in second-line treatment of advanced HER2+ disease compared to other anti-HER2 therapies, but their results are outdated ^24^^,^^25^. However, no systematic review has evaluated the use of T-DM1 in the treatment of early breast cancer (EBC) with residual invasive disease.

Given the lack of direct comparisons between these treatments versus T-DM1, a network meta-analysis (NMA) is a valid approach to assess their relative efficacy and toxicity. This systematic review and NMA aims to evaluate the clinical efficacy and safety of T-DM1 compared to other anti-HER2 therapies in patients with advanced HER2+ disease who respond to trastuzumab and taxanes, as well as in patients with early-stage disease who have pre-stage invasive residual disease after trastuzumab- and taxane-based neoadjuvant therapy.

**KEY MESSAGES**

Motivation for the study: Treatment options for HER2-positive breast cancer were evaluated, focusing on the efficacy and safety of trastuzumab-emtansine (T-DM1) compared to other anti-HER2 therapies.

Main findings: Trastuzumab-deruxtecan (T-DXd) and PyroCap emerged as promising alternatives, showing substantial improvements in progression-free survival for locally advanced or metastatic breast cancer. T-DM1 showed superior efficacy to the other treatments.

Implications: Our findings could inform healthcare decision-making processes to optimize strategies for HER2-positive breast cancer, and potentially improve health outcomes and quality of life.

## MATERIALS AND METHODS

This study followed the Cochrane Handbook of Systematic Reviews ^26^ and the PRISMA statement ^27^, together with the NMA statement ^28^ for reporting. The protocol was published in the International Prospective Register of Systematic Reviews (PROSPERO registry: CRD42021266771).

### Selection criteria

Types of studies: Completed Phase II and/or III randomised controlled trials (RCTs).

Types of participants: HER2+ BC patients (diagnosed by immunohistochemistry [IHC] or fluorescence *in situ* hybridization [FISH]/chromogenic *in situ* hybridization [CISH]), including EBC with residual disease after neoadjuvant treatment with trastuzumab and taxanes and surgery, unresectable locally advanced breast cancer (LABC) or metastatic breast cancer (mBC) with progression during or after more recent treatment with trastuzumab or biosimilar (≥ 80% of the cohort) and a taxane (in advanced disease or within six months after treatment of early-stage disease). Patients with low HER2+ expression, previous T-DM1 therapy (>20% of the cohort), patients with uncontrolled brain metastases, and lack of discriminate results for the disease of interest by disease stage or previous treatment were excluded.

Types of interventions: the intervention of interest corresponds to T-DM1 therapy and any other intervention that could be considered for the same indication (afatinib, atezolizumab, bevacizumab, capecitabine, everolimus, lapatinib, margetuximab, neratinib, pertuzumab, pirotinib, sunitinib, trastuzumab-deruxtecan, trastuzumab, tucatinib, vinorelbine and other immunotherapies or chemotherapies). Also, best supportive care or placebo were considered as comparators. Aromatase inhibitors (e.g. tamoxifen or toremifene) were acceptable co-interventions.

Measurement of outcomes: overall survival (OS), progression-free survival (PFS), objective response rate (ORR), invasive disease-free survival (iDFS), treatment-related adverse events (AEs) defined according to the Common Terminology Criteria for Adverse Events (CTCAE), including grade 3 (severe AEs) or higher (life-threatening or disabling), AEs leading to discontinuation of treatment (EAdisc) and serious AEs (SAEs).

### Search strategy and data source

An experienced librarian from the research group developed a sensitive, unfiltered, language-sensitive search strategy for articles published as of January 2018 (search date of the Paracha *et al.*^24^, sought to be updated) (Supplementary Material). We searched the following databases from 01/01/2018 to 5/05/2021: MEDLINE, EMBASE, LILACS, The Cochrane Library, CINAHL and Global Health. In addition, reference lists of all included studies and identified systematic reviews were reviewed. It was not necessary to obtain additional relevant evidence beyond that provided in the identified studies.

For studies with multiple publications, we considered the parent study or the larger sample study as a primary reference. Secondary references were used to supplement the data. An expert in the field has been consulted for the inclusion of additional studies with relevant information (Supplementary Material).

### Study selection and data collection

Selection, data extraction, and risk of bias assessment were performed independently by peer reviewers from the research team. Discrepancies were resolved by consensus of the entire team. All phases of study selection were carried out using COVIDENCE® ^29^^,^^30^, a web-based platform designed for the systematic review process. We extracted general RCT data, location of the study, RCT phase (II and/or III), characteristics of the participants, intervention and comparators, and efficacy and safety outcome data.

### Assessment of risk of bias

**Figure 1:**
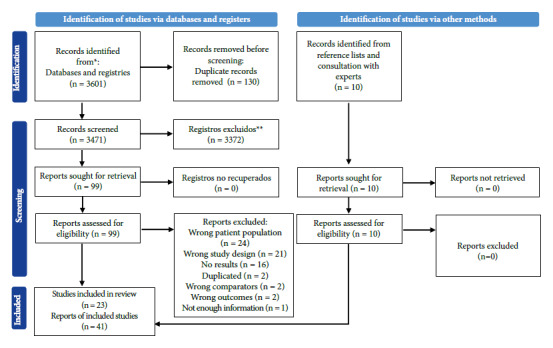
Flow chart of the study.

We independently assessed the risk of bias of included studies using the Cochrane risk of bias assessment ^31^. Within each domain (randomisation process, deviations from intended interventions, missing outcome data, outcome measurement, selection of reported outcome) and overall risk of bias, the flagging questions lead to judgements of “low risk of bias”, “some concerns” or “high risk of bias”. The results of the risk of bias assessment were communicated through graphs and tables summarising these findings (Figure 1 and Figure A1 in the Supplementary Material).

### Statistical analysis

An NMA using a random effects model was performed for each of the outcomes of interest using the “netmeta” package (version 2.0-1) of the free software R (version 4.0.5). For the interpretation of the results, the largest network was selected. For the efficacy results of the OS and PFS, the hazard ratio (HR) was used as a measure of effect. Where the HR was not available, data from risk tables and Kaplan-Meier curves were used to estimate it. Transitivity and consistency assumptions were assessed. Transitivity was evaluated based on the comparison of the populations of each study, comparing the distribution of potential effect modifiers, among the treatment comparisons. To verify the consistency assumption, two graphical tools, a net head plot, and a forest plot, were used, comparing direct and indirect evidence by using the loop-specific approach as described by Higgins *et al.*^32^.

The net heat plot provides two pieces of information, the inconsistency generated by one comparison (row) to another comparison (column) which is considered based on the background color, where the color red represents greater inconsistency and the white color less inconsistency. In turn, the size of the blocks determines how much a comparison (row) impacts the overall estimate of another comparison (column), where the larger the size indicates the greater the importance of the comparison (of the row) in the estimation of the comparison represented in the column. The degree of overlap between direct and indirect evidence and their directions of the same based on the no-effect line.

We presented the geometry of the network and the results in probability statements, as well as in forest diagrams. We assessed statistical heterogeneity using the I² statistic and considered I²= 30-60% values as an indication of “moderate” heterogeneity that justifies the use of the random-effects model for the synthesis of results. The assessment of network-wide statistical heterogeneity was based on the magnitude of the heterogeneity variance parameter (I²) estimated from the NMA models. We carried out subgroup analyses according to the stage of disease progression (EBC and LABC/mBC).

Our confidence in the estimates for each reported outcome was assessed using the Grading of Recommendations Assessment, Development, and Evaluation (GRADE) approach, including specific criteria for assessing confidence in NMA estimates, potential intransitivity (based on the potential effect-modifying variables described in the subgroup analysis), and potential inconsistency (based on the consistency assessment) ^33^^,^^34^.

## RESULTS

### Study Selection

We retrieved 3,471 non-duplicate records and we selected 109 potentially eligible articles for full-text review. Finally, 41 reports representing 23 studies were included (Figure 1).

The main baseline patient characteristics and study features are presented in Table 1. The studies included RCTs (fourteen phase-III and nine phase-II studies) analyzing 6,737 total participants. The range of median ages reported by the studies was between 48-60 years. Thirteen studies included patients with HER2+ mBC ^(20,35-47)^, eight patients analyzed LABC ^(11,48-56)^, and two studies included EBC ^12^^,^^57^.

**Table 1:** Patient and study characteristics.

Author, Year	RCT phase	Country	BC Stage	Randomized patients	Intervention arm (Sample size)	Control arm (Sample size)	Median age	Net	Risk of bias
Jiang 2019 (36)	III	Multicentre	mBC	279 (2:1)	PyroCap (185)	Cap (94)	NR	PFS, ORR	High Risk
Sim 2019 (37)	II	NR	mBC	149 (1:1)	LapVino (75)	Vino (74)	NR	X	High Risk
Ma 2019 (38)	II	Multicentre (China)	mBC	128 (1:1)	PryroCap (65)	LapCap (63)	48/49	OS, PFS, ORR, AEdisc, EA>3	Some Concerns
Urruticoechea 2017 (39,40)	III	Multicentre	mBC	452 (1:1)	PerTrasCap (228)	TrasCap (224)	54/55	OS, PFS, ORR, AEdisc	Some Concerns
Bischoff 2019 (41)	II	Multicentre (Germany)	mBC	43 (1:1)	LapEri 1.76mg/m2 (21)	LapEri1.23mg/m2 (22)	60/50	X	High Risk
Xu 2021(35)	III	Multicentre (China)	mBC	267 (1:1)	PyroCap (134)	LapCap (133)	50/49	PFS, ORR, AEdisc, AE>3	Low Risk
André 2014 (42)	III	Multicentre	mBC	569 (1:1)	EveroVinoTras (284)	VinoTras (285)	54.5/54	X	Some Concerns
Blackwell 2009 (43,59)	III	Multicentre	mBC	296 (1:1)	Lap (148)	LapTras (148)	51/52	X	Some Concerns
Johnston 2018 (44)	III	Multicentre	mBC	355 (1:1:1)	LapTras (120) /Lap (118)	Tras (117)	57/57/54	X	Some Concerns
Bian 2020 (45)	III	Multicentre (China)	mBC	315 (2:1)	CiptVino (212)	Vino (103)	50/49	X	Some Concerns
Cortés 2020 (46)	II	Multicentre	mBC	161 (1:1)	T-DM1Cap (81)	T-DM1 (80)	54/52	OS, PFS, ORR, AEdisc, SAEs, EA>3	High Risk
Harbeck 2016(47)	III	Multicentre	mBC	508 (2:1)	Afa (339)	Tras (169)	51.8/53.1	X	High Risk
Cortes J 2021(20,60)	III	Multicentre	mBC	524 (1:1)	T-DXd (261)	T-DM1 (263)	54.3/54.2	OS, PFS, ORR, AEdisc, SAEs, AE>3	High Risk
Martin 2013 (56)	II	Multicentre	LABC+ mBC	233 (1:1)	Nera (117)	LapCap (116)	52/56	OS, PFS, ORR, AEdisc, SAEs, AE>3	Some Concerns
Takano 2018(48)	II	Multicentre	LABC+ mBC	86 (1:1)	TrasCap (43)	LapCap (43)	53/59	OS, PFS, ORR, AEdisc	Some Concerns
Tolaney 2020 (49)	II	Multicentre	LABC+ mBC	237 (1:1:1)	AbemaTrasFulv (79)/AbemaTras (79)	ChemotherapyTras (79)	55/54/57	X	Low Risk
Verma 2012(11,50)	III	Multicentre	LABC+ mBC	991 (1:1)	T-DM1 (495)	LapCap (496)	53/53	OS, PFS, ORR, AEdisc, SAEs, AE>3	Some Concerns
Haddad 2021(51)	II	USA	LABC+ mBC	55 (1:2)	LapCap (19)	LapCapCitu (36)	NR	OS, PFS, AE>3	Some Concerns
Geyer 2006/Cameron 2008 (52,53)	III	Multicentre	LABC+ mBC	399 (1:1)	LapCap (198)	Cap (201)	54/51	OS, PFS, ORR, AEdisc, SAEs	Some Concerns
von Minckwitz 2009(54)	III	Multicentre	LABC+ mBC	156 (1:1)	TrasCap (78)	Cap (78)	59/52	OS, PFS, ORR, AEdisc	Some Concerns
Emens 2020 (55)	II	Multicentre	LABC+ mBC	202 (2:1)	T-DM1Atezo (133)	T-DM1 (69)	54/55	OS, PFS, ORR, AEdisc, SAEs	Low Risk
Harbeck 2020 (57)	III	NR	EBC	1846 (1:1)	T-DM1Pertu (923)	TrasPertuTax (923)	NR	X	High Risk
von Minckwitz 2019(12)	III	Multicentre	EBC	1486 (1:1)	T-DM1 (743)	Tras (743)	49/49	X	High Risk

X: not part of any network, mBC: metastatic breast cancer, LABC: local advance breast cancer, EBC: early breast cancer, OS: overall survival, PFS: progression free survival, ORR: overall rate ratio, AEdisc: adverse events discontinuation, SAEs: severe adverse events, NR: not reported.

### Risk-of-bias

About half of the studies had “some concern” for risk of bias. About 35% of the studies had a high risk of bias. Table 1 details the risk of bias assessment for each study and Figure A1 in the Supplementary Material shows the overall risk of bias ranking by domain.

 Summary of findings (SoF) tables were generated to compare the different treatments against T-DM1 for OS and PFS. They show the networks used, the meta-analysis estimates, and the quality of the evidence for each of these comparisons.

### Effect of interventions for patients with metastatic or locally advanced breast cancer

### Overall survival (OS)

Fifteen RCTs reported on OS. Eleven were included in the main network with 11 different treatments. The NMA included 55 comparisons, eight with direct evidence only, three with mixed evidence and the others with indirect evidence only (I^2^: 59.2%; 95% confidence interval [CI]: 0%-90.04%). No statistically significant differences were observed between any of the treatments (Table 2). The three comparisons with mixed evidence showed some inconsistency (Figure A2 and Figure A3 in the Supplementary Material).

**Table 2:** Cross-tabulation of treatment for overall survival.

	T-DM1	T-DXd	T-DM1Atezo	PerTrasCap	T-DM1Cap	PyroCap	TrasCap	LapCapCitu	LapCap	Cap	Nera
T-DM1		1.79 (0.83-3.86)	1.35 (0.58-3.17)	1.17 (0.36-3.81)	1.15 (0.47-2.82)	1.09 (0.38-3.14)	0.89 (0.34-2.33)	0.81 (0.26-2.51)	0.75 (0.39-1.43)	0.69 (0.29-1.66)	0.60 (0.22-1.62)
T-DXd			0.76 (0.24-2.39)	0.65 (0.16-2.68)	0.64 (0.20-2.10)	0.61 (0.16-2.26)	0.50 (0.14-1.71)	0.45 (0.12-1.78)	0.42 (0.15-1.15)	0.39 (0.12-1.24)	0.34 (0.10-1.18)
T-DM1Atezo				0.86 (0.20-3.71)	0.85 (0.25-2.93)	0.80 (0.21-3.13)	0.66 (0.18-2.38)	0.60 (0.15-2.47)	0.56 (0.19-1.62)	0.51 (0.15-1.73)	0.44 (0.12-1.64)
PerTrasCap					0.99 (0.22-4.35)	0.93 (0.25-3.41)	0.76 (0.38-1.5)	0.69 (0.18-2.69)	0.64 (0.24-1.73)	0.59 (0.23-1.49)	0.51 (0.15-1.78)
T-DM1Cap						0.95 (0.24-3.80)	0.77 (0.21-2.89)	0.70 (0.17-2.99)	0.65 (0.22-1.98)	0.60 (0.17-2.11)	0.52 (0.14-1.99)
PyroCap							0.82 (0.27-2.46)	0.75 (0.21-2.60)	0.69 (0.30-1.60)	0.63 (0.23-1.77)	0.55 (0.18-1.70)
TrasCap								0.91 (0.28-2.95)	0.85 (0.41-1.73)	0.78 (0.41-1.46)	0.68 (0.24-1.91)
LapCapCitu									0.93 (0.37-2.34)	0.85 (0.28-2.56)	0.74 (0.22-2.45)
LapCap										0.92 (0.51-1.66)	0.80 (0.38-1.70)
Cap											0.87 (0.33-2.28)
Nera											

Results are expressed as Hazard Ratio along with their 95% confidence intervals.

Hazard Ratios > 1.00 favour the column-defining treatments (i.e. T-DXd has better overall survival than T-DM1 and TrasCap has worse overall survival than T-DM1).

### Progression-free survival (PFS)

Twenty RCTs reported on PFS. Thirteen were included in the main network with 11 different treatments. The NMA included 55 comparisons, seven with direct evidence, five with mixed evidence and the rest with indirect evidence (I^2^: 0%; 95% CI: 0%-84%). T-DXd showed more efficacy than all other treatments for PFS. T-DM1 alone was more effective than LapCap (HR: 0.65; 95% CI: 0.55-0.77), TrasCap (HR: 0.65; 95% CI: 0.46-0.91), LapCapCitu (HR: 0.6; 95% CI: 0.33-1.10), Nera (HR: 0.55; 95% CI: 0.39-0.77) and Cap (HR: 0.37; 95% CI: 0.28-0.49), and less effective than T-DXd (HR: 3.57; 95% CI: 2.75-4.63) and PyroCap (HR: 1.82; 95% CI: 1.35-2.44) (Table 3). Two of the five comparisons with mixed evidence showed considerable inconsistency, Cap vs. TrasCap and LapCap vs. TrasCap (Figure A4 and Figure A5 of the Supplementary Material).

**Table 3:** Cross-tabulation of treatment for progression-free survival.

	T-DM1	T-DXd	PyroCap	T-DM1Atezo	T-DM1Cap	PerTrasCap	LapCap	TrasCap	LapCapCitu	Nera	Cap
T-DM1		3.57 (2.75-4.63)	1.82 (1.35-2.44)	1.22 (0.82-1.82)	1.09 (0.80-1.48)	0.78 (0.53-1.15)	0.65 (0.55-0.77)	0.65 (0.46-0.91)	0.60 (0.33-1.10)	0.55 (0.39-0.77)	0.37 (0.28-0.49)
T-DXd			0.51 (0.34-0.75)	0.34 (0.21-0.55)	0.30 (0.20-0.46)	0.22 (0.14-0.35)	0.18 (0.13-0.25)	0.18 (0.12-0.28)	0.17 (0.09-0.33)	0.15 (0.10-0.23)	0.10 (0.07-0.15)
PyroCap				0.67 (0.41-1.11)	0.60 (0.39-0.92)	0.43 (0.29-0.64)	0.36 (0.28-0.46)	0.36 (0.25-0.50)	0.33 (0.18-0.62)	0.30 (0.21-0.44)	0.20 (0.16-0.26)
T-DM1Atezo					0.89 (0.54-1.48)	0.64 (0.36-1.12)	0.53 (0.34-0.82)	0.53 (0.31-0.90)	0.50 (0.24-1.02)	0.45 (0.26-0.76)	0.30 (0.19-0.50)
T-DM1Cap						0.72 (0.43-1.18)	0.60 (0.42-0.85)	0.59 (0.38-0.94)	0.56 (0.28-1.09)	0.50 (0.32-0.80)	0.34 (0.22-0.52)
PerTrasCap							0.83 (0.59-1.19)	0.83 (0.68-1.02)	0.78 (0.40-1.53)	0.70 (0.44-1.11)	0.48 (0.34-0.67)
LapCap								0.99 (0.74-1.33)	0.93 (0.52-1.65)	0.84 (0.63-1.13)	0.57 (0.45-0.72)
TrasCap									0.94 (0.49-1.78)	0.85 (0.56-1.28)	0.57 (0.43-0.76)
LapCapCitu										0.90 (0.47; 1.72)	0.61 (0.33-1.14)
Nera											0.68 (0.47-0.98)
Cap											

Results are expressed as Hazard Ratio along with their 95% confidence intervals.

Hazard Ratios > 1.00 favour the column-defining treatments (i.e. T-DXd has a better progression-free survival than T-DM1 and TrasCap has a worse progression-free survival than T-DM1).

### Overall response rate (ORR)

Twenty RCTs reported ORR in patients with mBC or LABC, of which 12 were included in the NMA. Forty-five comparisons were made, of which six were from direct evidence, five from direct and indirect evidence, and the rest from indirect evidence only. T-DM1 showed a lower ORR than T-DXd (objective response [OR]: 0.13; 95% CI: 0.04-0.50) and the PyroCap combination (OR: 0.17; 95% CI: 0.04-0.82) (Figure A6 and Table A1 in Supplementary Material).

### Safety

The adverse effects and drug safety were evaluated in patients with mBC or LABC. A total of 16 RCTs assessed the discontinuation of treatment due to adverse effects (11 were included in the network), SAEs in 9 RCTs (6 were included in the network), and AEs ≥ grade 3 in 11 studies (7 were included in the network). 

For the analysis of AEdisc, 45 comparisons were made, of which seven came from direct evidence, three from direct and indirect evidence, and the rest from indirect evidence only. T-DM1 did not show statistically significant differences compared to the other treatments (Figure A7 and Table A2 of the Supplementary Material).

In terms of SAEs, 22 comparisons were made, of which six came from direct evidence and the rest from indirect evidence only. T-DM1 showed more SAEs compared to Cap (Odds ratio [OR]: 2.42; 95% CI: 1.32-4.43) and less SAEs compared to T-DM1Atezo (OR: 0.48; 95% CI: 0.24-0.96) (Figure A8 and Table A3 of the Supplementary Material).

For the analysis of grade ≥ 3 AEs, 21 comparisons were made, of which six came from direct evidence and the rest from indirect evidence only. T-DM1 showed a better profile compared to Neratinib (OR: 0.21; 95% CI: 0.11-0.43), PyroCap (OR: 0.24; 95% CI: 0.15-0.39) and LapCap (OR: 0.52; 95% CI: 0.40-0.67) (Figure A9 and Table A4 in the Supplementary Material). 

Other studies included in the review that were not included in the networks due to the lack of connection between the treatment arms and the network presented in this paper are listed in Table 1 ^37^^,^^41^^-^^45^^,^^47^^,^^49^.

### Effect of interventions for patients with early-stage breast cancer

Two studies evaluated the use of T-DM1 in EBC, but meta-analysis could not be performed due to immature data at the time of this review.

The KAITLIN study ^57^ showed in its preliminary data that, at three three-year follow-ups, there was no significant difference between the arms (T-DM1Pertu vs. TrasPertuTax) in the risk of events in the stratified invasive disease-free population (HR: 0.97; 95% CI: 0.71-1.32). Results were similar in the intention-to-treat population (HR: 0.98, 95% CI: 0.72-1.32). No OS data was available at the time of publication. The safety profile of T-DM1 was similar to the comparator arm.

The KATHERINE study ^12^ showed in its interim analysis that at 3 years the estimated percentage of patients free of invasive disease was 88.3% in the T-DM1 group and 77.0% in the trastuzumab group. Invasive disease-free survival was significantly higher in the T-DM1 group than in the trastuzumab group (HR: 0.50; 95% CI: 0.39-0.64; p<0.001). In terms of safety, there were more adverse events associated with T-DM1 than with trastuzumab alone.

## DISCUSSION

In the field of oncology, especially in the treatment of HER2+ advanced BC, T-DM1 has been the cornerstone of treatment ^58^. The aim of this study was to elucidate the changing landscape of therapeutic strategies for this cancer subtype, especially after progression following first-line treatments with taxanes and dual blockade with trastuzumab and pertuzumab. A systematic review and NMA of RCTs was conducted with the aim of providing a detailed overview of the comparative efficacy and safety between current and emerging treatment modalities.

The ascendance of T-DM1 as a preferred choice in the oncology community was significantly influenced by the findings of the EMILIA study (2012) ^11^. This pivotal trial underscored the superiority of T-DM1 over lapatinib, highlighting in particular a six-month survival benefit in patients who had previously progressed on taxanes and trastuzumab. Despite this, the therapeutic landscape has undergone a paradigm shift in recent years, with the introduction of new systemic treatment options that challenge the hegemony of T-DM1 as standard second-line therapy in advanced HER2+ BC. These new interventions include more potent anti-HER2 tyrosine kinase inhibitors (TKIs) such as neratinib, tucati-nib and pirotinib, the monoclonal antibody conjugate T-DXd, the anti-HER2 monoclonal antibody margetuximab, and immunotherapies such as atezolizumab.

Two systematic reviews with meta-analyses by Paracha *et al.* in 2020 ^24^ and Chen *et al.* in 2021 ^25^ addressed the same question as our study. Twenty-three randomised trials including the recently published Destiny-Breast 0322 with T-DXd were included in our study. We included 23 RCTs, including the notable Destiny-Breast 03 trial with T-DXd ^20^. Our NMA showed a trend towards greater efficacy of several treatments than T-DM1 in terms of OS, including T-DXd, T-DM1 combined with atezolizumab, and combinations of pertuzumab, trastuzumab and capecitabine, among others.

T-DXd showed statistically and clinically significant superiority over T-DM1 in terms of PFS, in both direct and indirect comparisons. In addition, combination therapies of T-DM1 with atezolizumab or capecitabine also show greater efficacy compared to T-DM1 alone. In contrast, T-DM1 maintains greater efficacy over combinations of lapatinib and capecitabine, lapatinib and trastuzumab, neratinib and capecitabine, and shows a trend towards greater PFS over the combination of pertuzumab, trastuzumab and capecitabine.

In terms of the ORR, T-DXd and the combination of pyrotinib and capecitabine, in that order, demonstrate statistically significant superiority over T-DM1. The Phase III Destiny Breast-03 study ^20^, which randomized 524 patients with previously progressed HER2+ advanced BC, further corroborates the efficacy of T-DXd over T-DM1 in terms of PFS and shows a trend towards improved survival at 12 months.

The role of immunotherapy in HER2+ BC is increasingly recognized. The Phase II KATE2 study ^55^, comparing T-DM1 with T-DM1 plus atezolizumab (anti-PD-L1), revealed a trend favoring the combination therapy in patients expressing PD-L1 in at least 1% of peritumoral immune cells. This finding suggests the potential benefits of this combination in a subset of patients with advanced HER2+ and PD-L1+ breast cancer.

Another combination showing a tendency for superior OS, but not PFS, compared to T-DM1 is pertuzumab, trastuzumab, and capecitabine. Despite its demonstrated benefits in early HER2+ disease and first-line advanced disease, this combination has not received regulatory approval due to the lack of significant difference in PFS in the Phase II PHEREXA study ^39^^,^^40^.

The safety profile of these therapeutic regimens forms an essential part of this analysis. However, the evaluation is somewhat constrained by the limited number of RCTs with complete data on discontinuation rates for study drug-related events and SAEs. Notably, only two therapeutic schemes with lower efficacy than T-DM1 tended to have lower discontinuation rates due to treatment-related toxicity: neratinib and the trastuzumab plus capecitabine combination. In the Destiny Breast 03 study ^20^, the treatment-related discontinuation rate for T-DXd was higher, primarily due to interstitial lung disease/pneumonitis, compared to T-DM1, which was mostly discontinued due to thrombocytopenia.

Regarding SAEs, only capecitabine showed a lower rate than T-DM1 in our meta-analysis. Other regimens reported a higher number of SAEs compared to T-DM1. In the Destiny Breast 03 study ^20^, the rate of grade 3 AE was higher with T-DXd, mainly due to myelotoxicity and gastrointestinal disorders, versus T-DM1, which predominantly caused thrombocytopenia and hepatotoxicity.

The Phase III PHENIX study ^36^ and the PHOEBE study ^35^ have also contributed significantly to the current understanding of these therapies. The PHENIX study demonstrated a statistically significant advantage in PFS for the combination of pyrotinib and capecitabine compared to capecitabine plus placebo. The PHOEBE study found that the combination of pyrotinib and capecitabine was more effective in median PFS (approximately six months). However, it is important to note that these studies with pyrotinib and capecitabine were conducted exclusively in the Chinese population, and the results should be ratified in other populations outside China. The last scheme with a tendency to be superior in our study in terms of OS, PFS, and ORR is the combination of T-DM1 and capecitabine. In the Phase II TRAXHER247 study, the T-DM1 and capecitabine arm did not prove to be better than T-DM1 monotherapy in the primary endpoint of the clinical trial, nor in PFS. This combination has also not been approved by regulatory bodies.

The strength of our systematic review and NMA lies in the possibility of capturing and analyzing all the available evidence, including the most recent RCTs (with low risk of bias) on the different treatment schemes in patients with advanced or locoregionally advanced HER2+ BC progressing to trastuzumab and taxanes as well as their reproducibility. On the other hand, it allowed the establishment of a therapeutic ranking based on the comparative efficacy and safety among the multiple interventions available. Briefly, T-DM1 presented a lower PFS, ORR, and tendency to lower OS than T-DXd, which dominated all the schemes studied in patients with advanced or locoregionally advanced HER2+ BC in patients previously progressed to trastuzumab and taxanes. In the safety analysis, on the other hand, T-DM1 was associated with a more favourable toxicity profile, with lower discontinuation rates due to drug-related events and fewer grade III/IV adverse events among the most effective treatment regimens.

The main limitations of this systematic review include the paucity of direct evidence between different comparisons of therapeutic regimens, which resulted in a limited evidence network and imprecision of the estimates, and also the presence of heterogeneity in several outcomes. On the other hand, the OS data may be strongly influenced by the availability and use of subsequent lines of treatment and the main toxicities of the treatments evaluated. While the central estimates of the analysis indicate variations in the efficacy of different treatments, the overall confidence in these findings is tempered by underlying uncertainties.

One of the key issues is the inclusion of clinical trials from various phases, which inherently possess different designs and objectives. This diversity in study design means that many direct comparisons between treatments are absent, underpowering the overall results and potentially skewing the results in an unknown direction. Additionally, despite being RCTs, some of these studies may harbor biases that could influence their outcomes, further complicating the interpretation of the meta-analysis. Specific patient characteristics and previous lines of treatment could influence the results. Another significant limitation is the temporal scope of the research. The search for relevant studies was conducted only up to May 5, 2021. Consequently, any developments or additional studies published after this date are not reflected in the analysis, potentially omitting crucial data that could affect the overall conclusions. Furthermore, there are concerns regarding compliance with certain assumptions inherent in NMA, such as transitivity. Transitivity assumes that the effects of treatments can be reliably compared indirectly through a common comparator across studies. However, if this assumption is not met in some comparisons, it could lead to questionable conclusions about the relative effectiveness of the treatments.

In conclusion, while T-DM1 remains a cornerstone in the treatment of advanced or locoregionally advanced HER2+ BC, especially following progression on trastuzumab and taxanes, it is now challenged by newer therapies like T-DXd. T-DXd has demonstrated superiority in PFS, ORR, and a tendency towards better OS. However, in terms of safety, T-DM1 exhibits a more favourable profile, with lower discontinuation rates due to drug-related events and fewer severe adverse events compared to the most effective treatment regimens. This evolving therapeutic landscape underscores the need for ongoing research and adaptation of treatment strategies in advanced HER2+ BC.
