# Supplementary material for: Trastuzumab-emtansine versus other anti-HER2 regimens in early or unresectable or metastatic HER-2 positive breast cancer: systematic review and network meta-analysis
Source: Rev Peru Med Exp Salud Publica. 2024 Mar 27;41(1):7–18. doi: 10.17843/rpmesp.2024.411.13351 (PMC11152244; doi:10.17843/rpmesp.2024.411.13351)
Supplement: Supplementary material. — Available in the electronic version of the RPMESP. [file rpmesp-41-01-13351-s001.pdf]

Appendix

Appendix 1. Search strategy

Medline - PubMed

Search	Query	Results
#29	#26 AND #27 Filters: from 2018/1/1 - 3000/12/12	1,265
#28	#26 AND #27	4,707
#27	((Randomized Controlled Trial[pt] OR Controlled Clinical Trial[pt] OR Randomized Controlled Trials[Mesh] OR Random Allocation[Mesh] OR Double-Blind Method[Mesh] OR Single-Blind Method[Mesh] OR Clinical Trial[pt] OR Clinical Trials[Mesh]) OR (Clinical Trial[tw]) OR ((Singl*[tw] OR Doubl*[tw] OR Trebl*[tw] OR Tripl*[tw]) AND (Mask*[tw] OR Blind*[tw])) OR (Placebos[Mesh] OR Placebo*[tw] OR Random*[tw] OR Research Design [mh:noexp]) NOT (Animals [Mesh] NOT Human[Mesh]))	1,927,018
#26	#10 AND #25	32,029
#25	#11 OR #12 OR #13 OR #14 OR #15 OR #16 OR #17 OR #18 OR #19 OR #20 OR #21 OR #22 OR #23 OR #24	50,338
#24	neu Gene*[tiab]	792
#23	Her2-neu[tiab]	3,456
#22	Her2neu[tiab]	5,835
#21	cerbB2[tiab]	3,395
#20	cerbB-2[tiab]	67
#19	Factor-Receptor 2[tiab]	12,377

#18	ErbB-2[tiab]	3,975
#17	Erb-B2[tiab]	723
#16	ErbB2[tiab]	8,526
#15	HER-Overexpressing[tiab]	7
#14	HER-Positive[tiab]	114
#13	HER-2[tiab]	7,719
#12	HER2[tiab]	32,095
#11	Genes, erbB-2[Mesh]	3,020
#10	#1 OR #2 OR #3 OR #4 OR #5 OR #6 OR #7 OR #8 OR #9	410,758
#9	Breast Neoplas*[tiab]	11,018
#8	Breast Carcinom*[tiab]	31,023
#7	Breast Cancer[tiab]	290,073
#6	Breast Tumo*[tiab]	24,643
#5	Mammary Carcinom*[tiab]	8,395
#4	Mammary Tumo*[tiab]	15,247
#3	Mammary Cancer[tiab]	3,231
#2	Mammary Neoplas*[tiab]	954

#1	Breast Neoplasms[Mesh]	303,581

EMBase
Embase <1974 to 2021 May 05>

1	exp breast tumor/	568434
2	(Mammary adj2 Neoplas*).ti,ab.	1275
3	(Mammary adj2 Cancer).ti,ab.	4169
4	(Mammary adj2 Tumo*).ti,ab.	19479
5	(Mammary adj2 Carcinom*).ti,ab.	10699
6	(Breast adj2 Tumo*).ti,ab.	43087
7	(Breast adj2 Cancer).ti,ab.	421364
8	(Breast adj2 Carcinom*).ti,ab.	43864
9	(Breast adj2 Neoplas*).ti,ab.	2581
10	or/1-9	631841
11	exp proto oncogene/	17231
12	HER2.ti,ab.	58056
13	HER-2.ti,ab.	13258
14	HER-Positive.ti,ab.	210
15	HER-Overexpressing.ti,ab.	10
16	ErbB2.ti,ab.	11608
17	Erb-B2.ti,ab.	859
18	ErbB-2.ti,ab.	4531
19	"Factor-Receptor 2".ti,ab.	15891
20	cerbB-2.ti,ab.	128
21	cerbB2.ti,ab.	174
22	Her2neu.ti,ab.	554
23	Her2-neu.ti,ab.	5651
24	neu Gene*.ti,ab.	961
25	or/11-24	104245
26	10 and 25	62298
27	(random$ or placebo$).ti,ab.	1789741
28	((single$ or double$ or triple$ or treble$) and (blind$ or mask$)).ti,ab.	288756
29	controlled clinical trial$.ti,ab.	37394
30	RETRACTED ARTICLE/	11411
31	or/27-30	1859824
32	(animal$ not human$).sh,hw.	4539204
33	31 not 32	1685281
34	26 and 33	6310
35	limit 34 to yr="2018 -Current"	2102

CINAHL (EBSCO)

#	Query	Results
S27	S25 AND S26<br>Limiters - Published Date: 20180101-20210531;	329

S26	(MH "Clinical Trials+") OR PT Clinical trial OR TX clinic* n1 trial* OR TX ((singl* n1 blind*) OR (singl* n1 mask*)) OR TX ((doubl* n1 blind*) OR (doubl* n1 mask*)) OR TX ((tripl* n1 blind*) OR (tripl* n1 mask*)) OR TX ((trebl* n1 blind*) OR (trebl* n1 mask*))	918,545
S25	S10 AND S24	4,212
S24	S11 OR S12 OR S13 OR S14 OR S15 OR S16 OR S17 OR S18 OR S19 OR S20 OR S21 OR S22 OR S23	5,590
S23	TI (neu N2 Gene*) OR AB (neu N2 Gene*)	16
S22	TI Her2-neu OR AB Her2-neu	23
S21	TI Her2neu OR AB Her2neu	26
S20	TI cerbB2 OR AB cerbB2	12
S19	TI cerbB-2 OR AB cerbB-2	5
S18	TI "Factor-Receptor 2" OR AB "Factor-Receptor 2"	1,872
S17	TI ErbB-2 OR AB ErbB-2	58
S16	TI Erb-B2 OR AB Erb-B2	55
S15	TI ErbB2 OR AB ErbB2	567
S14	TI HER-Overexpressing OR AB HER-Overexpressing	2
S13	TI HER-Positive OR AB HER-Positive	51
S12	TI HER-2 OR AB HER-2	698
S11	TI HER2 OR AB HER2	3,907
S10	S1 OR S2 OR S3 OR S4 OR S5 OR S6 OR S7 OR S8 OR S9	64,173
S9	TI (Breast N1 Neoplas*) OR AB (Breast N1 Neoplas*)	234
S8	TI (Breast N1 Carcinom*) OR AB (Breast N1 Carcinom*)	3,009
S7	TI (Breast N1 Cancer) OR AB (Breast N1 Cancer)	75,334

S6	TI (Breast N1 Tumo*) OR AB (Breast N1 Tumo*)	3,318
S5	TI (Mammary N1 Carcinom*) OR AB (Mammary N1 Carcinom*)	337
S4	TI (Mammary N1 Tumo*) OR AB (Mammary N1 Tumo*)	667
S3	TI (Mammary N1 Cancer) OR AB (Mammary N1 Cancer)	200
S2	TI (Mammary N1 Neoplas*) OR AB (Mammary N1 Neoplas*)	33
S1	(MH "Breast Neoplasms+")	87,444

Cochrane Library (Wiley)
ID Search Hits
#1 MeSH descriptor: [Breast Neoplasms] explode all trees 13440
#2 (Mammary NEAR/1 Neoplas*):ti,ab,kw 12
#3 (Mammary NEAR/1 Cancer):ti,ab,kw 54
#4 (Mammary NEAR/1 Tumo*):ti,ab,kw 40
#5 (Mammary NEAR/1 Carcinom*):ti,ab,kw 93
#6 (Breast NEAR/1 Tumo*):ti,ab,kw 1576
#7 (Breast NEAR/1 Cancer*):ti,ab,kw 35120
#8 (Breast NEAR/1 Carcinom*):ti,ab,kw 1587
#9 (Breast NEAR/1 Neoplas*):ti,ab,kw 13901
#10 #1 OR #2 OR #3 OR #4 OR #5 OR #6 OR #7 OR #8 OR #9 37589
#11 MeSH descriptor: [Genes, erbB-2] explode all trees 40
#12 HER2:ti,ab,kw 5749
#13 HER-2:ti,ab,kw 778
#14 HER-Positive:ti,ab,kw 12
#15 (HER NEAR/2 Overexpressing):ti,ab,kw 28
#16 ErbB2:ti,ab,kw 271
#17 Erb-B2:ti,ab,kw 166
#18 ErbB-2:ti,ab,kw 900
#19 "Factor-Receptor 2":ti,ab,kw 2325
#20 cerbB-2:ti,ab,kw 50
#21 cerbB2:ti,ab,kw 29
#22 Her2neu:ti,ab,kw 213
#23 "Her2-neu":ti,ab,kw 242
#24 (neu NEAR/2 Gene*):ti,ab,kw 27
#25 #11 OR #12 OR #13 OR #14 OR #15 OR #16 OR #17 OR #18 OR #19 OR #20 OR #21 OR #22 OR #23 OR #24 7013
#26 #10 AND #25 with Publication Year from 2018 to 2021, with Cochrane Library publication date Between Jan 2018 and May 2021, in Trials 1852

LILACS (BVS Eng)
(MH Breast Neoplasms OR ((Breast OR Mammary OR Mama OR Pecho OR Seio OR Mamari$) AND (Cancer OR Tumor$ OR Neoplas$ OR Carcinom$))) AND (MH Genes, erbB-2 OR HER2 OR HER-2 OR HER-Positiv$ OR ErbB2 OR Erb-B2 OR cerbB-2 OR cerbB2 OR Her2neu OR Her2-neu OR neu-Gene$) [Words] and 2018

OR 2019 OR 2020 OR 2021 [Country, year publication] and (PT Ensayo Clínico Controlado Aleatorio OR PT Ensayo Clínico Controlado OR MH Ensayo Clínico Controlado Aleatorio OR MH Distribución Aleatoria OR MH Método Doble Ciego OR MH Método Simple-Ciego OR PT Ensayo Clínico OR MH Ensayo Clínico OR ((clinical OR clínico) AND (Ensaio OR Trial OR Ensayo)) OR ((singl$ OR simpl$ OR doubl$ OR trebl$ OR tripl$) AND (Random$ OR Aleatori$))) [Words]

2 [refine]

Global Health (OVID)

Global Health <1910 to 2021 Week 17>

1	exp Breast Neoplasms/	30300
2	(Mammary adj2 Neoplas*).ti,ab.	151
3	(Mammary adj2 Cancer).ti,ab.	547
4	(Mammary adj2 Tumo*).ti,ab.	1737
5	(Mammary adj2 Carcinom*).ti,ab.	527
6	(Breast adj2 Tumo*).ti,ab.	1526
7	(Breast adj2 Cancer).ti,ab.	29516
8	(Breast adj2 Carcinom*).ti,ab.	1645
9	(Breast adj2 Neoplas*).ti,ab.	145
10	or/1-9	36353
11	HER2.ti,ab.	985
12	HER-2.ti,ab.	412
13	HER-Positive.ti,ab.	16
14	HER-Overexpressing.ti,ab.	0
15	ErbB2.ti,ab.	234
16	Erb-B2.ti,ab.	23
17	ErbB-2.ti,ab.	106
18	"Factor-Receptor 2".ti,ab.	582
19	cerbB-2.ti,ab.	12
20	cerbB2.ti,ab.	7
21	Her2neu.ti,ab.	6
22	Her2-neu.ti,ab.	147
23	neu Gene*.ti,ab.	18
24	or/11-23	1989
25	10 and 24	1298
26	limit 25 to yr="2018 -Current"	329

Appendix 2

Figure A1: Figure Risk of bias

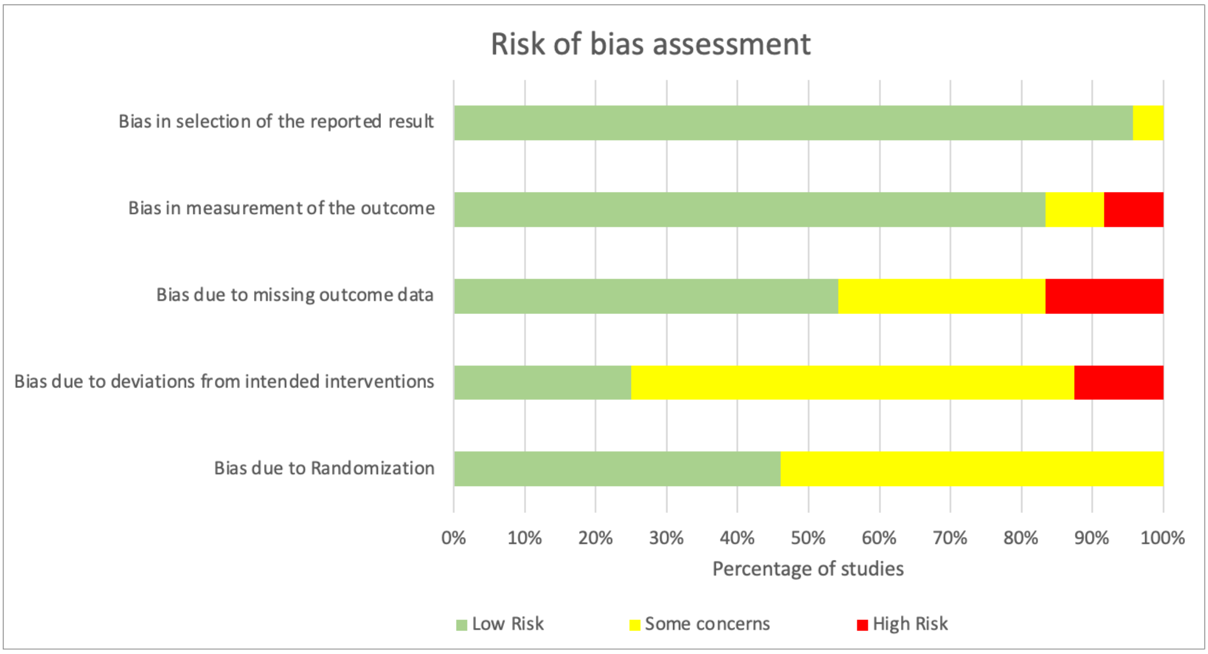

Figure A2: Net heat plot for OS

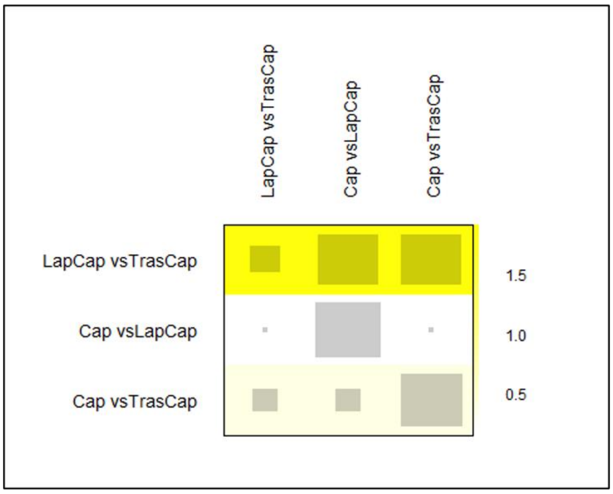

Figure A3: Net splitting for OS

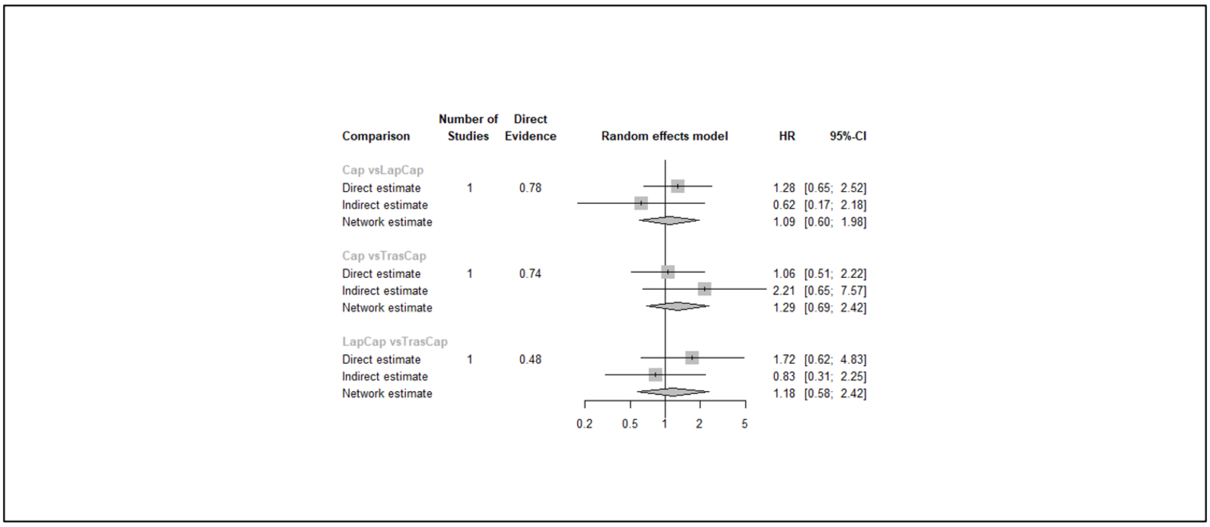

Figure A4: Net heat plot for PFS

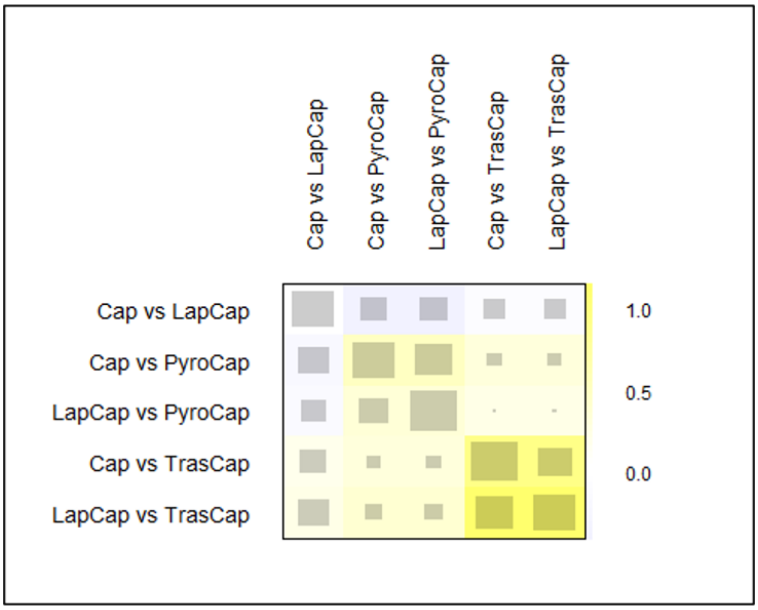

Figure A5: Net splitting for PFS

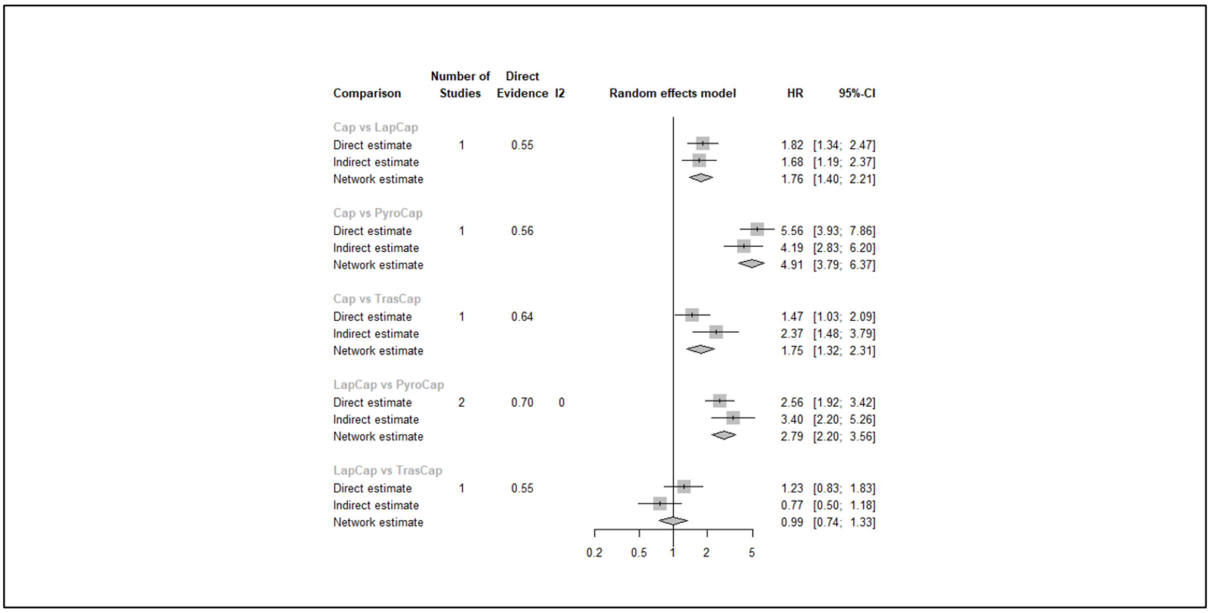

Figure A6: Treatment network plot for ORR

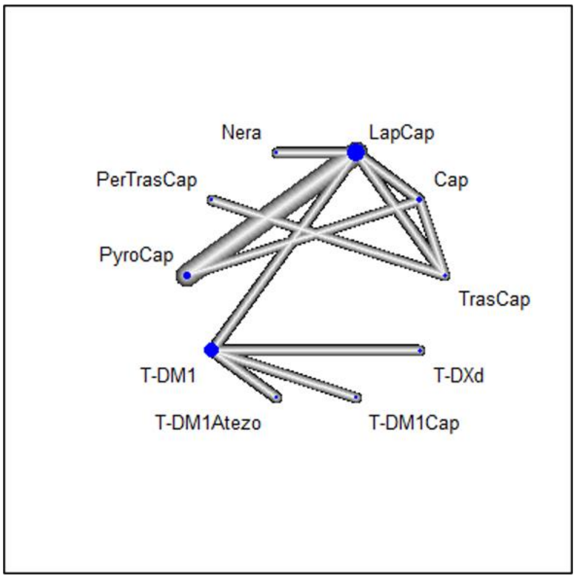

Table A1. Cross tabulation of treatment for ORR- OR (95% CI)

	T-DM1	T-DXd	PyroCap	LapCap	T-DM1Cap	Nera	PerTrasCap	T-DM1Atezo	Cap	TrasCap
**T-DM1**		0.13 (0.04; 0.5)	0.17 (0.04; 0.82)	0.54 (0.15; 1.98)	0.71 (0.17; 2.92)	0.9 (0.14; 5.95)	0.92 (0.1; 8.28)	0.92 (0.23; 3.71)	1.02 (0.2; 5.09)	1.27 (0.22; 7.28)
**T-DXd**			1.29 (0.17; 9.98)	4.08 (0.64; 25.97)	5.36 (0.78; 37.08)	6.78 (0.68; 68.04)	6.9 (0.53; 90.08)	6.96 (1.02; 47.47)	7.68 (0.96; 61.63)	9.57 (1.07; 85.62)
**PyroCap**				3.17 (1.32; 7.61)	4.17 (0.51; 34.24)	5.27 (1.03; 26.9)	5.36 (0.8; 35.85)	5.41 (0.67; 43.87)	5.96 (2.12; 16.78)	7.43 (1.93; 28.57)
**LapCap**					1.31 (0.19; 8.92)	1.66 (0.42; 6.57)	1.69 (0.29; 10.04)	1.71 (0.25; 11.42)	1.88 (0.72; 4.89)	2.35 (0.73; 7.58)
**T-DM1Cap**						1.27 (0.12; 13.37)	1.29 (0.09; 17.61)	1.3 (0.18; 9.42)	1.43 (0.17; 12.18)	1.78 (0.19; 16.87)
**Nera**							1.02 (0.11; 9.65)	1.03 (0.1; 10.72)	1.13 (0.21; 6.03)	1.41 (0.23; 8.59)
**PerTrasCap**								1.01 (0.07; 13.65)	1.11 (0.19; 6.46)	1.39 (0.36; 5.29)
**T-DM1Atezo**									1.1 (0.13; 9.25)	1.37 (0.15; 12.83)
**Cap**										1.25 (0.4; 3.89)
**TrasCap**										

ORs < 1.00 are interpreted against the treatment mentioned as the header in the row (i.e: T-DM1 has 87% less overall response compared to T-DXd, while it has 27% more overall response compared to TrasCap).

Figure A7: Treatment network plot for discontinuation due to AEs.

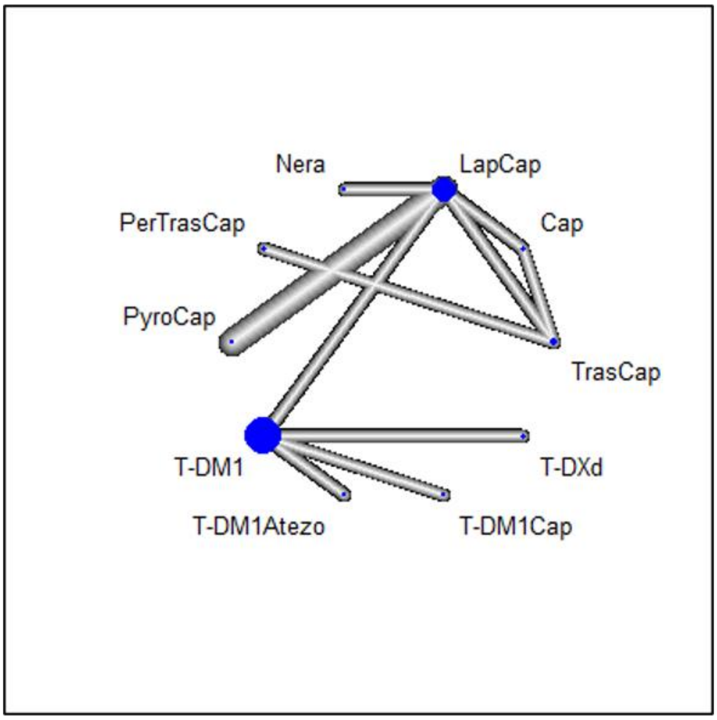

Table A2: Cross tabulation of treatment for AEdisc - OR (95% CI)

	T-DM1	Nera	TrasCap	PerTrasCap	Cap	LapCap	PyroCap	T-DM1Atezo	T-DXd	T-DM1Cap
T-DM1		2.15 (0.47; 9.77)	1.09 (0.28; 4.28)	0.98 (0.19; 4.96)	0.9 (0.26; 3.05)	0.67 (0.28; 1.65)	0.59 (0.13; 2.7)	0.5 (0.17; 1.46)	0.44 (0.17; 1.16)	0.35 (0.12; 1)
Nera			0.51 (0.1; 2.52)	0.46 (0.07; 2.83)	0.42 (0.09; 1.84)	0.31 (0.09; 1.07)	0.27 (0.05; 1.56)	0.23 (0.04; 1.5)	0.21 (0.03; 1.24)	0.16 (0.03; 1.03)
TrasCap				0.9 (0.37; 2.16)	0.82 (0.3; 2.24)	0.62 (0.22; 1.74)	0.54 (0.11; 2.69)	0.46 (0.08; 2.61)	0.4 (0.08; 2.15)	0.32 (0.06; 1.79)
PerTrasCap					0.92 (0.24; 3.48)	0.69 (0.18; 2.68)	0.6 (0.1; 3.77)	0.51 (0.07; 3.59)	0.45 (0.07; 2.98)	0.36 (0.05; 2.47)
Cap						0.75 (0.33; 1.75)	0.66 (0.15; 2.92)	0.56 (0.11; 2.85)	0.49 (0.1; 2.34)	0.39 (0.08; 1.96)
LapCap							0.87 (0.25; 2.99)	0.74 (0.18; 3)	0.65 (0.18; 2.43)	0.52 (0.13; 2.06)
PyroCap								0.85 (0.13; 5.48)	0.75 (0.12; 4.54)	0.59 (0.09; 3.77)
T-DM1Atezo									0.88 (0.21; 3.71)	0.69 (0.15; 3.12)
T-DXd										0.79 (0.19; 3.29)
T-DM1Cap										

ORs > 1.00 are interpreted against the treatment listed as the row heading (i.e: TDM1 has 115% more treatment discontinuation than neratinib, while T-DM1 has a 56% reduced risk of discontinuation compared to T-DXd)

Figure A8: Treatment network plot for Serious Adverse Events (SAE)

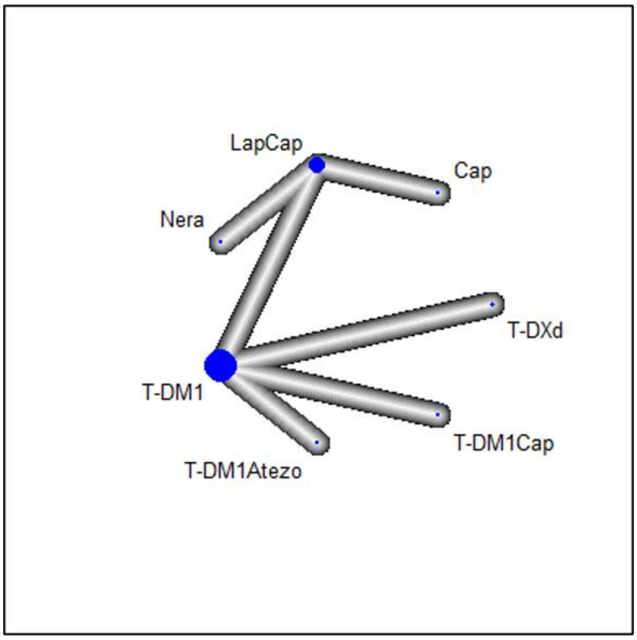

Table A3: Cross tabulation of treatment for SAE- OR (95% CI)

	T-DM1	Cap	T-DM1Cap	LapCap	Nera	T-DXd	T-DM1Atezo
**T-DM1**		2.42 (1.32; 4.43)	0.95 (0.38; 2.39)	0.84 (0.6; 1.17)	0.64 (0.32; 1.27)	0.53 (0.28; 1.01)	0.48 (0.24; 0.96)
**Cap**			0.39 (0.13; 1.18)	0.35 (0.21; 0.57)	0.26 (0.12; 0.58)	0.22 (0.09; 0.53)	0.2 (0.08; 0.5)
**T-DM1Cap**				0.88 (0.33; 2.35)	0.67 (0.21; 2.12)	0.56 (0.18; 1.72)	0.5 (0.16; 1.6)
**LapCap**					0.76 (0.42; 1.39)	0.64 (0.31; 1.31)	0.57 (0.26; 1.24)
**Nera**						0.83 (0.33; 2.14)	0.75 (0.28; 2)
**T-DXd**							0.9 (0.35; 2.33)
**T-DM1Atezo**							

ORs < 1.00 are interpreted in favour of the treatment listed as the row heading (i.e: T-DM1 has a higher rate of serious adverse events compared to Cap, while T-DM1 has a lower rate of serious adverse events compared to T-DXd)

Figure A9: Treatment network plot for Adverse Effect ≥ grade 3 (AES3)

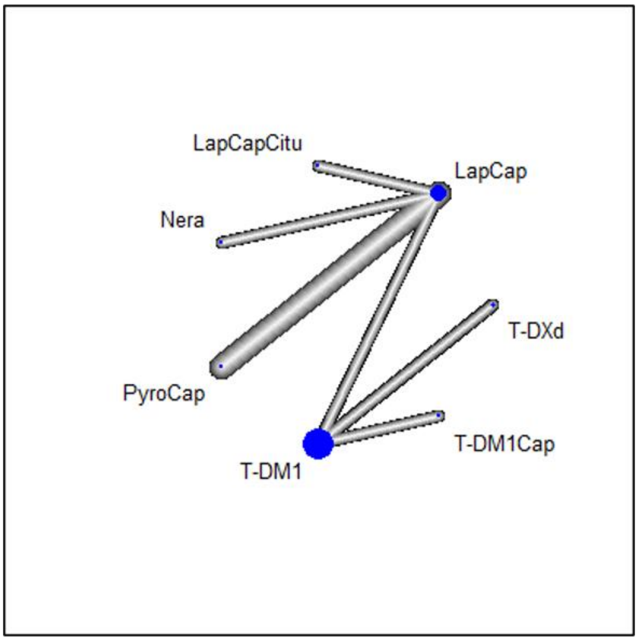

Table A4: Cross tabulation of treatment for AES3- OR (95% CI)

	T-DM1	T-DM1Cap	T-DXd	LapCap	LapCapCitu	PyroCap	Nera
T-DM1		0.88 (0.47; 1.66)	0.86 (0.61; 1.21)	0.52 (0.4; 0.67)	0.46 (0.14; 1.45)	0.24 (0.15; 0.39)	0.21 (0.11; 0.43)
T-DM1Cap			0.97 (0.47; 1.99)	0.59 (0.3; 1.16)	0.51 (0.14; 1.92)	0.28 (0.13; 0.61)	0.24 (0.09; 0.62)
T-DXd				0.61 (0.39; 0.93)	0.53 (0.16; 1.78)	0.28 (0.16; 0.51)	0.25 (0.11; 0.54)
LapCap					0.88 (0.28; 2.71)	0.47 (0.31; 0.7)	0.41 (0.22; 0.79)
LapCapCitu						0.54 (0.16; 1.78)	0.47 (0.13; 1.73)
PyroCap							0.88 (0.41; 1.88)
Nera							

ORs < 1.00 are interpreted in favour of the treatment listed as the row header (i.e: T-DM1 has 12% fewer grade ≥ 3 adverse events tha
