# Supplementary material for: Trastuzumab-emtansine versus other anti-HER2 regimens in early or unresectable or metastatic HER-2 positive breast cancer: systematic review and network meta-analysis
Source: Rev Peru Med Exp Salud Publica. 2024 Mar 27;41(1):7–18. doi: 10.17843/rpmesp.2024.411.13351 (PMC11152244; doi:10.17843/rpmesp.2024.411.13351)
Supplement: Translation

# Translation: Trastuzumab-emtansina versus otros regímenes anti-HER-2 en el cáncer de mama HER-2 positivo precoz o irresecable o metastásico: revisión sistemática y metaanálisis en red

**Authors:** Agustín Ciapponi, Ariel Bardach, Carla Colaci, Federico Rodríguez Cairoli, Fernando Argento, Ernesto Korbenfeld, Sebastián García Martí

**Affiliations:** 1 Instituto de Efectividad Clínica y Sanitaria, Buenos Aires, Argentina.; 2 Health Technology Assessment and Health Economics Department of the Institute for Clinical Effectiveness and Health Policy (IECS-CONICET), Buenos Aires, Argentina.; 3 Hospital Británico de Buenos Aires, Buenos Aires, Argentina.

**Keywords:** Genes HER-2, Cáncer de Mama, Metaanálisis en Red, Revisión Sistemática, Trastuzumab Emtansina

## Abstract

**Objetivo.:**

Nuestro objetivo fue estudiar la eficacia y la seguridad de trastuzumab-emtansina (T-DM1) en comparación con otras terapias anti-HER-2 en el cáncer de mama (CM) HER-2 positivo.

**Materiales y métodos.:**

Realizamos un metaanálisis de red (NMA, por sus siglas en inglés) de ensayos clínicos aleatorizados (ECA). Nuestro estudio se centró en pacientes sometidos al tratamiento para el cáncer de mama localmente avanzado no resecable (CMLA) o cáncer de mama metastásico (CMm), que incluía esquemas con trastuzumab y taxanos. Además, consideramos casos dentro de los primeros 6 meses de tratamiento para el cáncer de mama temprano (CMT) HER-2 positivo.

**Resultados.:**

Se incluyeron en nuestro análisis un total de 23 ECA y 41 reportes. En CMLA y CMm, no se observaron diferencias estadísticamente significativas en ninguna de las comparaciones entre T-DM1 y otras terapias anti-HER-2 positivo. Al evaluar la sobrevida libre de progresión (SLP), trastuzumab-deruxtecan (T-DXd) y PyroCap demostraron una mayor eficacia en comparación con otros tratamientos (Hazard Ratio [HR]: 3,57; intervalo de confianza al 95% [IC 95%]: 2,75-4,63 y HR: 1.82; IC 95%: 1,35-2,44; respectivamente), mientras que T-DM1 por sí solo mostró una efectividad superior en comparación con LapCap (HR: 0,65; IC 95%: 0,55-0,77), TrasCap (HR: 0,65; IC 95%: 0,46-0,91), LapCapCitu (HR: 0,60; IC 95%: 0,33-1,1), Nera (HR: 0,55; IC 95%: 0,39-0,77) y Cap (HR: 0,37; IC 95%: 0,28-0,49).

**Conclusiones.:**

Este NMA estableció un ranking basado en la eficacia y seguridad comparativas entre las intervenciones disponibles. Aunque superior a otros esquemas, T-DM1 mostró una menor eficacia en la SLP y la tasa de respuesta objetiva, y una tendencia hacia una sobrevida global peor que T-DXd.

## INTRODUCCIÓN

En todo el mundo, el cáncer de mama (CM) es el cáncer más frecuente y la principal causa de muerte relacionada con el cáncer en las mujeres. Cada año, se diagnostican más de dos millones de casos y es responsable de más de 600 000 muertes en todo el mundo. Aproximadamente, la mitad de los casos y el 60% de las muertes se producen en países en vías de desarrollo ^1^.

La selección del tipo, esquema y secuencia de tratamiento depende de la extensión de la enfermedad y de diversos factores clínicos, siendo una consideración crucial, la sobreexpresión del receptor 2 del factor de crecimiento epidérmico humano (HER-2+) ^2^. El cáncer de mama (CM) HER-2+ se caracteriza por un comportamiento agresivo, que se traduce en una menor sobrevida global (SG) y sin enfermedad, tanto en estadios tempranos como avanzados. La aprobación del trastuzumab (Herceptin®) en 1998 cambió el paradigma del tratamiento, haciendo hincapié en la prolongación de la sobrevida libre de progresión (SLP) ^3^^-^^5^ y de la SG ^6^^-^^8^. En 2006, la aprobación de la *Food and Drug Administration* de Estados Unidos (FDA) amplió el uso de trastuzumab en la enfermedad en estadio inicial basándose en los beneficios demostrados para la sobrevida libre de enfermedad (SLD) y la SG en grandes ensayos de fase III ^7^^,^^9^^,^^10^.

En los últimos años, se han incorporado nuevos fármacos anti-HER-2 con mecanismos diversos, como anticuerpos monoclonales, inhibidores de la tirosina cinasa y anticuerpos monoclonales conjugados como trastuzumab-emtansina (T-DM1) y trastuzumab-deruxtecan (T-DXd). T-DM1 (Kadcyla®), el primer anticuerpo monoclonal conjugado, obtuvo la aprobación reglamentaria en 2013 para el cáncer de mama metastásico o no resecable locorregionalmente avanzado. Su aprobación por la FDA (noviembre de 2013) y la *European Medicines Agency* (EMA) supuso un avance significativo en el tratamiento del CM HER-2+ avanzado basado en los resultados del estudio pivotal EMILIA ^11^. En mayo de 2019, la FDA aprobó T-DM1 como tratamiento adyuvante de la enfermedad precoz HER-2+ en pacientes con enfermedad residual invasiva persistente tras un tratamiento neoadyuvante basado en trastuzumab y taxanos, como se demostró en el ensayo clínico KATHERINE ^12^.

Investigaciones recientes han explorado terapias anti-HER-2, ampliando las indicaciones de T-DM1 para la enfermedad avanzada HER-2+ (previamente tratada y progresada con trastuzumab y taxanos). Entre ellos, se incluyen anticuerpos monoclonales dirigidos contra el dominio extracelular de HER-2 ^6^^-^^8^^,^^13^^,^^14^, inhibidores de moléculas pequeñas de la actividad tirosina cinasa de los receptores de la familia HER ^15^^-^^19^ y anticuerpos monoclonales conjugados recientemente aprobados como trastuzumab-deruxtecan (T-DXd) ^20^. Los estudios también han explorado combinaciones con quimioterápicos como capecitabina ^21^ y vinorelbina ^22^, así como fármacos diana específicos como everolimus (inhibidor de mTOR) ^23^. Revisiones sistemáticas y metaanálisis anteriores han evaluado la eficacia y la seguridad de T-DM1 en el tratamiento de segunda línea de la enfermedad HER-2+ avanzada en comparación con otros tratamientos anti-HER-2, pero sus resultados están desfasados ^24^^,^^25^. Sin embargo, ninguna revisión sistemática evaluó el uso de T-DM1 en el tratamiento del cáncer de mama precoz con enfermedad invasiva residual.

Dada la falta de comparaciones directas entre estos tratamientos frente a T-DM1, un metaanálisis en red (NMA, por sus siglas en inglés) es un enfoque válido para evaluar su eficacia y toxicidad relativas. Esta revisión sistemática y meta-análisis en red tiene como objetivo evaluar la eficacia clínica y la seguridad de T-DM1 en comparación con otras terapias anti-HER-2 en pacientes con enfermedad HER-2+ avanzada que responden a trastuzumab y taxanos, así como en pacientes con enfermedad en estadio temprano que presentan enfermedad residual invasiva tras la terapia neoadyuvante basada en trastuzumab y taxanos.

**MENSAJE CLAVE**

Motivación para realizar el estudio: Se evaluaron las opciones de tratamiento para el cáncer de mama HER-2-positivo, centrándose en la eficacia y seguridad de trastuzumab-emtansina (T-DM1) en comparación con otras terapias anti-HER-2.

Principales hallazgos: Trastuzumab-deruxtecan (T-DXd)y PyroCap surgieron como alternativas prometedoras, mostrando mejoras sustanciales en la sobrevida libre de progresión para el cáncer de mama localmente avanzado o metastásico. T-DM1 mostró una eficacia superior a la de los demás tratamientos.

Implicancias: Nuestros hallazgos podrían informar los procesos de toma de decisiones sanitarias para optimizar las estrategias para el cáncer de mama HER-2-positivo, y potencialmente mejorar los resultados de salud y la calidad de vida.

## MATERIALES Y MÉTODOS

Este estudio siguió el *Cochrane Handbook of Systematic Reviews*^26^ y la declaración PRISMA ^27^, junto con la extensión para metaanálisis en red ^28^ para la elaboración de informes. El protocolo se publicó en el *International Prospective Register of Systematic Reviews* (registro PROSPERO: CRD42021266771).

### Criterios de selección

Tipos de estudios: Ensayos controlados aleatorizados (ECA) de fase II y/o III completados. 

Tipos de participantes: pacientes con cáncer de mama HER-2+ (diagnóstico por Inmunohistoquímica [IHC, por sus siglas inglés] o técnica *fluorescence in situ hybridization* [FISH] /*chromogenic in situ hybridization* [CISH]), incluyendo cáncer de mama precoz (CMP) con enfermedad residual tras tratamiento neoadyuvante con trastuzumab y taxanos y cirugía, cáncer de mama localmente avanzado (CMLA) no resecable o cáncer de mama metastásico (CMm), con progresión durante o después de un tratamiento más reciente con trastuzumab o biosimilar (≥ 80% de la cohorte) y un taxano (en enfermedad avanzada o en los seis meses siguientes al tratamiento de la enfermedad en estadio precoz). Se excluyeron aquellas pacientes que tenían baja expresión HER-2+, que tenían terapias previas con T-DM1 (>20% de la cohorte), pacientes con metástasis cerebrales no controladas y ausencia de resultados discriminados para la enfermedad de interés según el estadio de la enfermedad o el tratamiento previo.

Tipos de intervenciones: la intervención de interés corresponde a la terapia con T-DM1 y cualquier otra intervención que pudiera considerarse para la misma indicación (afatinib, atezolizumab, bevacizumab, capecitabina, everolimus, lapatinib, margetuximab, neratinib, pertuzumab, pirotinib, sunitinib, trastuzumab-deruxtecan, trastuzumab, tucatinib, vinorelbina y otras inmunoterapias o quimioterapias). También, se consideraron mejores cuidados de apoyo o placebo como comparadores. Los inhibidores de la aromatasa (p. ej., tamoxifeno o toremifeno) fueron cointervenciones aceptables.

Medición de desenlaces: sobrevida global (SG), sobrevida libre de progresión (SLP), tasa de respuesta objetiva (TRO), sobrevida libre de enfermedad invasiva (SLEI), eventos adversos relacionados con el tratamiento (EA) definidos según los criterios terminológicos comunes para los eventos adversos (CTCAE, por sus siglas en inglés), incluidos los de grado 3 (EA severos) o superior (potencialmente mortales o incapacitantes), los EA que provocan la interrupción del tratamiento (iEA) y los EA graves (EAG).

### Estrategia de búsqueda y fuente de datos

Un bibliotecario experimentado del grupo de investigadores desarrolló una estrategia de búsqueda sensible, no filtrada, por idioma, de artículos publicados a partir de enero de 2018 (fecha de búsqueda del articulo o revisión Paracha *et al.*^24^, que se busca actualizar) (Material Suplementario). Se realizaron búsquedas en las siguientes bases de datos desde el 01/01/2018 hasta el 5/05/2021: MEDLINE, EMBASE, LILACS, The Cochrane Library, CINAHL y Global Health. Además, se revisaron las listas de referencias de todos los estudios incluidos y las revisiones sistemáticas identificadas. No fue necesario obtener pruebas relevantes adicionales a las proporcionadas en los estudios identificados.

Para los estudios con múltiples publicaciones, se consideró el estudio principal o el estudio de muestra más amplio como referencia primaria. Se utilizaron referencias secundarias para complementar los datos. Se consultó a un experto en la materia para la inclusión de estudios adicionales con información relevante (Material Suplementario).

### Selección de estudios y recopilación de datos

La selección, la extracción de datos y la evaluación del riesgo de sesgo fueron realizadas de forma independiente por dos revisores del equipo de investigación. Las discrepancias se resolvieron por consenso de todo el equipo. Todas las fases de selección de los estudios se llevaron a cabo utilizando COVIDENCE® ^29^^,^^30^, una plataforma web diseñada para el proceso de revisión sistemática. Se extrajeron los datos generales del ECA, la localización del estudio, la fase del ECA (II y/o III), las características de los participantes, la intervención y los comparadores, y los datos de los resultados de eficacia y seguridad.

### Evaluación del riesgo de sesgo

Se evaluó de forma independiente el riesgo de sesgo de los estudios incluidos mediante la evaluación Cochrane del riesgo de sesgo ^31^. Dentro de cada dominio (proceso de aleatorización, desviaciones de las intervenciones previstas, datos de resultados faltantes, medición del resultado, selección del resultado informado) y el riesgo general de sesgo, las preguntas de señalización conducen a juicios de «bajo riesgo de sesgo», «algunas preocupaciones» o «alto riesgo de sesgo». Los resultados de la evaluación del riesgo de sesgo se comunicaron mediante gráficos y tablas que resumían estos hallazgos (Figura 1 y Figura A1 del Material Suplementario).

**Figura 1:** Flujograma del estudio.

### Análisis estadístico

Se realizó un NMA utilizando un modelo de efectos aleatorios para cada uno de los resultados de interés mediante el paquete «netmeta» (versión 2.0-1) del software libre R (versión 4.0.5). Para la interpretación de los resultados, se seleccionó la red más extensa. Para los resultados de eficacia de la SG y la SLP, se utilizó el hazard ratio (HR) como medida del efecto. En los casos en los que el HR no estaba disponible, se utilizaron datos de tablas de riesgo y curvas de Kaplan-Meier para estimarla. Se evaluaron los supuestos de transitividad y consistencia. La transitividad se evaluó a partir de la comparación de las poblaciones de cada estudio, comparando la distribución de los modificadores potenciales del efecto, entre las comparaciones de tratamientos. Para verificar el supuesto de consistencia, se utilizaron dos herramientas gráficas, un *net head plot*, y *forest plot*, comparando la evidencia directa e indirecta mediante el uso del enfoque específico del bucle descrito por Higgins *et al.*^32^.

El *net head plot* proporciona dos informaciones, la incoherencia generada por una comparación (fila) respecto a otra comparación (columna, que se considera en función del color de fondo, donde el color rojo representa mayor incoherencia y el color blanco menor incoherencia. A su vez, el tamaño de los bloques determina cuánto influye una comparación (fila) en la estimación global de otra comparación (columna). Cuanto mayor es el tamaño, mayor es la importancia de la comparación (de la fila) en la estimación de la comparación representada en la columna. El grado de solapamiento entre las pruebas directas e indirectas y sus direcciones se basa en la línea sin efecto.

Presentamos la geometría de la red y los resultados en declaraciones de probabilidad, así como en *forest diagrams*. Se evaluó la heterogeneidad estadística mediante el estadístico I² y se consideraron los valores de I² de 30-60% como una indicación de heterogeneidad «moderada» que justifica el uso del modelo de efectos aleatorios para la síntesis de los resultados. La evaluación de la heterogeneidad estadística en toda la red se basó en la magnitud del parámetro de varianza de la heterogeneidad (I²) estimada a partir de los modelos NMA. Se realizaron análisis por subgrupos en función del estadio de progresión de la enfermedad (CMP y CMLA/CMm).

Nuestra confianza en las estimaciones para cada resultado notificado se evaluó mediante el enfoque GRADE (*Grading of Recommendations Assessment, Development, and Evaluation*), que incluye criterios específicos para evaluar la confianza en las estimaciones de los NMA, la posible intransitividad (basada en las posibles variables modificadoras del efecto descritas en el análisis por subgrupos) y la posible inconsistencia (basada en la evaluación de la consistencia) ^33^^,^^34^.

## RESULTADOS

### Selección de estudios

Se recuperaron 3471 registros no duplicados y se seleccionaron 109 artículos potencialmente elegibles para la revisión a texto completo. Finalmente, se incluyeron 41 informes que representaban 23 estudios (Figura 1).

Las principales características basales de los pacientes y las características de los estudios se presentan en la Tabla 1. Los estudios incluyeron ECA (catorce estudios de fase III y nueve de fase II) que analizaron un total de 6737 participantes. La mediana de edad comunicada por los estudios oscilaba entre 48 y 60 años. Trece estudios incluyeron pacientes con cáncer de mama metastásico HER-2+ ^20^^,^^35^^-^^47^, ocho estudios analizaron CMP ^11^^,^^48^^-^^56^ y dos estudios incluyeron CMP ^12^^,^^57^.

**Tabla 1:** Características de los pacientes y del estudio.

Autor, año	Fase del ECA	País	Estadio del CM	Pacientes aleatorizados	Brazo de intervención (tamaño de muestra)	Brazo de control (tamaño de muestra)	Edad (Mediana)	Red	Riesgo de sesgo
Jiang, 2019 ^36^	III	Multicéntrico	CMm	279 (2:1)	PyroCap (185)	Cap (94)	NR	SLP	Alto riesgo
Sim, 2019 ^37^	II	NR	CMm	149 (1:1)	LapVino (75)	Vino (74)	NR	X	Alto riesgo
Ma, 2019 ^38^	II	Multicéntrico (China)	CMm	128 (1:1)	PryroCap (65)	LapCap (63)	48/49	SG, SLP, TRO, iEA, EA>3	Algunas preocupaciones
Urruticoechea, 2017 ^39^^,^^40^	III	Multicéntrico	CMm	452 (1:1)	PerTrasCap (228)	TrasCap (224)	54/55	SG, SLP, TRO, iEA	Algunas preocupaciones
Bischoff, 2019 ^41^	II	Multicéntrico (Alemania)	CMm	43 (1:1)	LapEri 1.76mg/m^2^ (21)	LapEri1.23mg/m^2^ (22)	60/50	X	Alto riesgo
Xu, 2021^35^	III	Multicéntrico (China)	CMm	267 (1:1)	PyroCap (134)	LapCap (133)	50/49	SLP, TRO, iEA, EA>3	Bajo riesgo
André, 2014 ^42^	III	Multicéntrico	CMm	569 (1:1)	EveroVinoTras (284)	VinoTras (285)	54.5/54	X	Algunas preocupaciones
Blackwell, 2009 ^43^^,^^59^	III	Multicéntrico	CMm	296 (1:1)	Lap (148)	LapTras (148)	51/52	X	Algunas preocupaciones
Johnston 2018 (44)	III	Multicéntrico	CMm	355 (1:1:1)	LapTras (120) /Lap (118)	Tras (117)	57/57/54	X	Algunas preocupaciones
Bian, 2020 ^45^	III	Multicéntrico (China)	CMm	315 (2:1)	CiptVino (212)	Vino (103)	50/49	X	Algunas preocupaciones
Cortés, 2020 ^46^	II	Multicéntrico	CMm	161 (1:1)	T-DM1Cap (81)	T-DM1 (80)	54/52	SG, SLP, TRO, iEA, EAG, EA>3	Alto riesgo
Harbeck, 2016 ^47^	III	Multicéntrico	CMm	508 (2:1)	Afa (339)	Tras (169)	51,8/53,1	X	Alto riesgo
Cortes J, 2021 ^20^^,^^60^	III	Multicéntrico	CMm	524 (1:1)	T-DXd (261)	T-DM1 (263)	54,3/54,2	SG, SLP, TRO, iEA, EAG, EA>3	Alto riesgo
Martin, 2013 ^56^	II	Multicéntrico	CMLA + CMm	233 (1:1)	Nera (117)	LapCap (116)	52/56	SG, SLP, TRO, iEA, EAG, EA>3	Algunas preocupaciones
Takano, 2018 ^48^	II	Multicéntrico	CMLA + CMm	86 (1:1)	TrasCap (43)	LapCap (43)	53/59	SG, SLP, TRO, iEA	Algunas preocupaciones
Tolaney, 2020 ^49^	II	Multicéntrico	CMLA + CMm	237 (1:1:1)	AbemaTrasFulv (79)/ AbemaTras (79)	ChemotherapyTras (79)	55/54/57	X	Bajo riesgo
Verma, 2012 ^11^^,^^50^	III	Multicéntrico	CMLA + CMm	991 (1:1)	T-DM1 (495)	LapCap (496)	53/53	SG, SLP, TRO, iEA, EAG, EA>3	Algunas preocupaciones
Haddad, 2021 ^51^	II	Multicéntrico	CMLA + CMm	55 (1:2)	LapCap (19)	LapCapCitu (36)	NR	SG, SLP, EA>3	Algunas preocupaciones
Geyer , 2006/Cameron, 2008 ^52^^,^^53^	III	Multicéntrico	CMLA + CMm	399 (1:1)	LapCap (198)	Cap (201)	54/51	SG, SLP, TRO, iEA, EAG	Algunas preocupaciones
von Minckwitz, 2009 ^54^	III	Multicéntrico	CMLA + CMm	156 (1:1)	TrasCap (78)	Cap (78)	59/52	SG, SLP, TRO, iEA, EAG	Algunas preocupaciones
Emens, 2020 ^55^	II	Multicéntrico	CMLA + CMm	202 (2:1)	T-DM1Atezo (133)	T-DM1 (69)	54/55	SG, SLP, TRO, iEA, EAG	Bajo riesgo
Harbeck, 2020 ^57^	III	NR	CMm	1846 (1:1)	T-DM1Pertu (923)	TrasPertuTax (923)	NR	X	Alto riesgo
von Minckwitz, 2019 ^12^^)^	III	Multicéntrico	CMm	1486 (1:1)	T-DM1 (743)	Tras (743)	49/49	X	Alto riesgo

X: no forma parte de ninguna red, CMm: cáncer de mama metastásico; CMLA: cáncer de mama local avanzado; CMP: cáncer de mama precoz; SG: sobrevida global; SLP: sobrevida libre de progresión; TRO: tasa de respuesta objetivo; EA: eventos adversos, iEA: interrupción por eventos adversos; EAG: eventos adversos graves

### Riesgo de sesgo

Aproximadamente, la mitad de los estudios tenían «cierta preocupación» por el riesgo de sesgo. Alrededor del 35% de los estudios presentaban un alto riesgo de sesgo. La Tabla 1 detalla la evaluación del riesgo de sesgo para cada estudio y la Figura A1 del Material Suplementario muestra la clasificación general del riesgo de sesgo por dominio.

Se generaron tablas de resumen de resultados (SoF, por sus siglas en inglés) para comparar los diferentes tratamientos frente a T-DM1 para la SG y la SLP. Estas tablas muestran las redes utilizadas, las estimaciones del metaanálisis y la calidad de la evidencia para cada una de estas comparaciones.

### Efecto de las intervenciones en pacientes con cáncer de mama metastásico o localmente avanzado

### Sobrevida global

Quince ECA informaron sobre la SG. Once se incluyeron en la red principal con 11 tratamientos diferentes. El metaanálisis en red incluyó 55 comparaciones, ocho sólo con evidencia directa, tres con evidencia mixta y las demás, sólo con evidencia indirecta (I^2^: 59,2%; intervalo de confianza al 95% [IC 95%]: 0%-90,04%). No se observaron diferencias estadísticamente significativas entre ninguno de los tratamientos (Tabla 2). Las tres comparaciones con evidencia mixta mostraron cierta inconsistencia (Figura A2 y Figura A3 del Material Suplementario).

**Tabla 2:** Tabulación cruzada del tratamiento para la sobrevida global.

	T-DM1	T-DXd	T-DM1Atezo	PerTrasCap	T-DM1Cap	PyroCap	TrasCap	LapCapCitu	LapCap	Cap	Nera
T-DM1		1,79 (0,83-3,86)	1,35 (0,58-3,17)	1,17 (0,36-3,81)	1,15 (0,47-2,82)	1,09 (0,38-3,14)	0,89 (0,34-2,33)	0,81 (0,26-2,51)	0,75 (0,39-1,43)	0,69 (0,29-1,66)	0,60 (0,22-1,62)
T-DXd			0,76 (0,24-2,39)	0,65 (0,16-2,68)	0,64 (0,20-2,10)	0,61 (0,16-2,26)	0,50 (0,14-1,71)	0,45 (0,12-1,78)	0,42 (0,15-1,15)	0,39 (0,12-1,24)	0,34 (0,10-1,18)
T-DM1Atezo				0,86 (0,20-3,71)	0,85 (0,25-2,93)	0,80 (0,21-3,13)	0,66 (0,18-2,38)	0,60 (0,15-2,47)	0,56 (0,19-1,62)	0,51 (0,15-1,73)	0,44 (0,12-1,64)
PerTrasCap					0,99 (0,22-4,35)	0,93 (0,25-3,41)	0,76 (0,38-1,5)	0,69 (0,18-2,69)	0,64 (0,24-1,73)	0,59 (0,23-1,49)	0,51 (0,15-1,78)
T-DM1Cap						0,95 (0,24-3,80)	0,77 (0,21-2,89)	0,70 (0,17-2,99)	0,65 (0,22-1,98)	0,60 (0,17-2,11)	0,52 (0,14-1,99)
PyroCap							0,82 (0,27-2,46)	0,75 (0,21-2,60)	0,69 (0,30-1,60)	0,63 (0,23-1,77)	0,55 (0,18-1,70)
TrasCap								0,91 (0,28-2,95)	0,85 (0,41-1,73)	0,78 (0,41-1,46)	0,68 (0,24-1,91)
LapCapCitu									0,93 (0,37-2,34)	0,85 (0,28-2,56)	0,74 (0,22-2,45)
LapCap										0,92 (0,51-1,66)	0,80 (0,38-1,70)
Cap											0,87 (0,33-2,28)
Nera											

Los resultados están expresados como Hazard Ratio junto con sus intervalos de confianza al 95%.

Los Hazard Ratio > 1,00 favorecen a los tratamientos que definen la columna (es decir, T-DXd presenta una mejor sobrevida global que T-DM1 y TrasCap una peor sobrevida global que T-DM1).

### Sobrevida libre de progresión

Veinte ECA informaron sobre la SLP. Trece se incluyeron en la red principal con 11 tratamientos diferentes. El meta-análisis en red incluyó 55 comparaciones, siete con evidencia directa, cinco con evidencia mixta y las demás, con evidencia indirecta (I^2^: 0%; IC 95%: 0%-84%). T-DXd mostró más eficacia que todos los demás tratamientos para la SLP. T-DM1 solo fue más eficaz que LapCap (HR: 0,65; IC 95%: 0,55-0,77), TrasCap (HR: 0,65; IC 95%: 0,46-0,91), LapCapCitu (HR: 0,6; IC 95%: 0,33-1,10), Nera (HR: 0,55; IC 95%: 0,39-0,77) y Cap (HR: 0,37; IC 95%: 0,28-0,49), y menos eficacia que T-DXd (HR: 3,57; IC 95%: 2,75-4,63) y PyroCap (HR: 1,82; IC 95%: 1,35-2,44) (Tabla 3). Dos de las cinco comparaciones con evidencia mixta mostraron una inconsistencia considerable, Cap *vs.* TrasCap y LapCap *vs.* TrasCap (Figura A4 y Figura A5 del Material Suplementario).

**Tabla 3:** Tabulación cruzada del tratamiento para la sobrevida libre de progresión.

	T-DM1	T-DXd	PyroCap	T-DM1Atezo	T-DM1Cap	PerTrasCap	LapCap	TrasCap	LapCapCitu	Nera	Cap
T-DM1		3,57 (2,75-4,63)	1,82 (1,35-2,44)	1,22 (0,82-1,82)	1,09 (0,80-1,48)	0,78 (0,53-1,15)	0,65 (0,55-0,77)	0,65 (0,46-0,91)	0,60 (0,33-1,10)	0,55 (0,39-0,77)	0,37 (0,28-0,49)
T-DXd			0,51 (0,34-0,75)	0,34 (0,21-0,55)	0,30 (0,20-0,46)	0,22 (0,14-0,35)	0,18 (0,13-0,25)	0,18 (0,12-0,28)	0,17 (0,09-0,33)	0,15 (0,10-0,23)	0,10 (0,07-0,15)
PyroCap				0,67 (0,41-1,11)	0,60 (0,39-0,92)	0,43 (0,29-0,64)	0,36 (0,28-0,46)	0,36 (0,25-0,50)	0,33 (0,18-0,62)	0,30 (0,21-0,44)	0,20 (0,16-0,26)
T-DM1Atezo					0,89 (0,54-1,48)	0,64 (0,36-1,12)	0,53 (0,34-0,82)	0,53 (0,31-0,90)	0,50 (0,24-1,02)	0,45 (0,26-0,76)	0,30 (0,19-0,50)
T-DM1Cap						0,72 (0,43-1,18)	0,60 (0,42-0,85)	0,59 (0,38-0,94)	0,56 (0,28-1,09)	0,50 (0,32-0,80)	0,34 (0,22-0,52)
PerTrasCap							0,83 (0,59-1,19)	0,83 (0,68-1,02)	0,78 (0,40-1,53)	0,70 (0,44-1,11)	0,48 (0,34-0,67)
LapCap								0,99 (0,74-1,33)	0,93 (0,52-1,65)	0,84 (0,63-1,13)	0,57 (0,45-0,72)
TrasCap									0,94 (0,49-1,78)	0,85 (0,56-1,28)	0,57 (0,43-0,76)
LapCapCitu										0,90 (0,47; 1,72)	0,61 (0,33-1,14)
Nera											0,68 (0,47-0,98)
Cap											

Los resultados están expresados como Hazard Ratio junto con sus intervalos de confianza al 95%.

Los Hazard Ratio > 1,00 favorecen a los tratamientos que definen la columna (es decir, T-DXd presenta una sobrevida libre de progresión mejor que T-DM1 y TrasCap una sobrevida libre de progresión peor que T-DM1).

### Tasa de respuesta objetiva

Veinte ECA informaron de la TRO en pacientes con CMm o CMLA, de los cuales 12 se incluyeron en el metaanálisis en red. Se realizaron 45 comparaciones, de las cuales 6 procedían de pruebas directas, 5 de pruebas directas e indirectas y el resto sólo de pruebas indirectas. T-DM1 mostró una tasa de TRO menor que T-DXd (respuesta objetiva [RO]: 0,13; IC 95%: 0,04-0,50) y que la combinación PyroCap (RO: 0,17; IC 95%: 0,04-0,82). (Figura A6 y Tabla A1 del Material Suplementario).

### Seguridad

Se evaluaron los eventos adversos y la seguridad del fármaco en pacientes con CMm o CMLA. Un total de 16 ECA evaluaron los iEA (11 se incluyeron en la red), EAG en 9 ECA (6 se incluyeron en la red) y eventos adversos ≥ grado 3 en 11 estudios (7 se incluyeron en la red).

Para el análisis de iEA se realizaron 45 comparaciones, de las cuales siete procedían de pruebas directas, tres de pruebas directas e indirectas, y el resto sólo de pruebas indirectas. T-DM1 no mostró diferencias estadísticamente significativas en comparación con los demás tratamientos (Figura A7 y Tabla A2 del Material Suplementario).

En cuanto a los EAG, se realizaron 22 comparaciones, de las cuales seis procedían de pruebas directas y el resto sólo de pruebas indirectas. T-DM1 mostró más EAG en comparación con Cap (Odds ratio [OR]: 2,42; IC 95%: 1,32-4,43) y menos EAG en comparación con T-DM1Atezo (OR: 0,48; IC 95%: 0,24-0,96) (Figura A8 y Tabla A3 del Material Suplementario).

Para el análisis de los EA de grado ≥3, se realizaron 21 comparaciones, de las cuales seis procedían de evidencia directa y el resto sólo de evidencia indirecta. T-DM1 mostró un mejor perfil en comparación con Neratinib (OR: 0,21; IC 95%: 0,11-0,43), PyroCap (OR: 0,24; IC 95%: 0,15-0,39) y LapCap (OR: 0,52; IC 95%: 0,40-0,67) (Figura A9 y Tabla A4 del Material Suplementario).

Otros estudios incluidos en la revisión que no se incluyeron en las redes debido a la falta de conexión entre los brazos de tratamiento y la red presentada en este trabajo son presentados en la Tabla 1 ^37^^,^^41^^-^^45^^,^^47^^,^^49^.

### Efecto de las intervenciones para pacientes con cáncer de mama en estadio temprano

Dos estudios evaluaron el uso de T-DM1 en el CMP, pero no se pudo realizar el metaanálisis debido a la inmadurez de los datos en el momento de esta revisión.

El estudio KAITLIN ^57^ mostró en sus datos preliminares que, a los tres seguimientos de tres años, no hubo diferencias significativas entre los brazos (T-DM1Pertu frente a TrasPertuTax) en el riesgo de eventos en la población estratificada libre de enfermedad invasiva (HR: 0,97; IC 95%: 0,71-1,32). Los resultados fueron similares en la población por intención de tratar (HR: 0,98; IC 95%: 0,72-1,32). No se disponía de datos de SG en el momento de la publicación. El perfil de seguridad de T-DM1 fue similar al del brazo de comparación.

El estudio KATHERINE ^12^ mostró en su análisis provisional que, a los 3 años, el porcentaje estimado de pacientes libres de enfermedad invasiva era del 88,3% en el grupo de T-DM1 y del 77,0% en el grupo de trastuzumab. La sobrevida libre de enfermedad invasora fue significativamente mayor en el grupo de T-DM1 que en el de trastuzumab (HR: 0,50; IC 95%: 0,39-0,64; p<0,001). En cuanto a la seguridad, hubo más acontecimientos adversos asociados a T-DM1 que a trastuzumab solo.

## DISCUSIÓN

En el campo de la oncología, especialmente en el tratamiento del cáncer de mama avanzado HER-2+, el T-DM1 ha sido la piedra angular del tratamiento ^58^. El objetivo de este estudio era dilucidar el panorama cambiante de las estrategias terapéuticas para este subtipo de cáncer, especialmente después de la progresión tras los tratamientos de primera línea con taxanos y el bloqueo dual con trastuzumab y pertuzumab. Se llevó a cabo una revisión sistemática y un NMA de ECA con el objetivo de proporcionar una visión detallada de la eficacia y la seguridad comparativas entre las modalidades de tratamiento actuales y emergentes.

Los resultados del estudio Emilia (2012) influyeron significativamente en el ascenso de T-DM1 como opción preferida en la comunidad oncológica ^11^. Este ensayo fundamental subrayó la superioridad de T-DM1 frente a lapatinib, destacando en particular un beneficio de sobrevida de seis meses en pacientes que habían progresado previamente con taxanos y trastuzumab. A pesar de ello, el panorama terapéutico ha experimentado un cambio de paradigma en los últimos años, con la introducción de nuevas opciones de tratamiento sistémico que desafían la hegemonía de T-DM1 como terapia estándar de segunda línea en el cáncer de mama HER-2+ avanzado. Estas nuevas intervenciones incluyen inhibidores de la tirosina quinasa (TKI, por sus siglas en inglés) anti-HER-2 más potentes, como neratinib, tucatinib y pirotinib, el conjugado de anticuerpos monoclonales T-DXd, el anticuerpo monoclonal anti-HER-2 margetuximab e inmunoterapias como atezolizumab.

Dos revisiones sistemáticas con metaanálisis realizadas por Paracha *et al.* en 2020 ^24^ y Chen *et al.* en 2021 ^25^ abordaron la misma cuestión que nuestro estudio. En nuestro estudio se incluyeron 23 ensayos aleatorizados, incluido el recientemente publicado Destiny-Breast 0322 con T-DXd. Se incluyeron 23 ECA, incluido el notable ensayo Destiny-Breast 03 con T-DXd ^20^. Nuestro NMA mostró una tendencia hacia una mayor eficacia de varios tratamientos que T-DM1 en términos de SG, incluidos T-DXd, T-DM1 combinado con atezolizumab y combinaciones de pertuzumab, trastuzumab y capecitabina, entre otros.

T-DXd mostró una superioridad estadística y clínicamente significativa sobre T-DM1 en términos de SLP, en comparaciones directas e indirectas. Además, las terapias combinadas de T-DM1 con atezolizumab o capecitabina también muestran una mayor eficacia en comparación con T-DM1 solo. Por el contrario, T-DM1 mantiene una mayor eficacia frente a las combinaciones de lapatinib y capecitabina, lapatinib y trastuzumab, neratinib y capecitabina, y muestra una tendencia a una mayor SLP frente a la combinación de pertuzumab, trastuzumab y capecitabina.

En cuanto a la TRO, T-DXd y la combinación de pirotinib y capecitabina, en ese orden, demuestran una superioridad estadísticamente significativa sobre T-DM1. El estudio de fase III Destiny Breast-03 ^20^, que aleatorizó a 524 pacientes con cáncer de mama avanzado HER-2+ previamente progresado, corrobora aún más la eficacia de T-DXd sobre T-DM1 en términos de SLP y muestra una tendencia a la mejora de la sobrevida a los 12 meses.

Cada vez se reconoce más el papel de la inmunoterapia en el cáncer de mama HER-2+. El estudio de fase II KATE2 ^55^, que comparó T-DM1 con T-DM1 más atezolizumab (anti-PD-L1), reveló una tendencia favorable a la terapia combinada en pacientes que expresaban PD-L1 en al menos el 1% de las células inmunitarias peritumorales. Este hallazgo sugiere los beneficios potenciales de esta combinación en un subgrupo de pacientes con cáncer de mama avanzado HER-2+ y PD-L1+.

Otra combinación que muestra una tendencia a una SG superior, pero no a una SLP, en comparación con T-DM1 es pertuzumab, trastuzumab y capecitabina. A pesar de sus beneficios demostrados en la enfermedad HER-2+ temprana y en la enfermedad avanzada de primera línea, esta combinación no ha recibido la aprobación reglamentaria debido a la falta de diferencias significativas en la SLP en el estudio de fase II PHEREXA ^39^^,^^40^.

El perfil de seguridad de estos regímenes terapéuticos constituye una parte esencial de este análisis. Sin embargo, la evaluación se ve algo limitada por el escaso número de ECA con datos completos sobre las tasas de interrupción por EA relacionados con el fármaco en estudio y EAG. En particular, sólo dos esquemas terapéuticos con menor eficacia que T-DM1 tendieron a tener menores tasas de interrupción por toxicidad relacionada con el tratamiento: neratinib y la combinación de trastuzumab más capecitabina. En el estudio Destiny Breast 03 ^20^, la tasa de interrupción relacionada con el tratamiento de T-DXd fue mayor, principalmente debido a enfermedad pulmonar intersticial/neumonitis, en comparación con T-DM1, que se interrumpió principalmente debido a trombocitopenia.

En cuanto a los EAG, sólo capecitabina mostró una tasa inferior a T-DM1 en nuestro metaanálisis. Otros regímenes notificaron un mayor número de EAG en comparación con T-DM1. En el estudio Destiny Breast 03 ^20^, la tasa de EA de grado ≥3 fue mayor con T-DXd, debido principalmente a mielotoxicidad y trastornos gastrointestinales, frente a T-DM1, que causó predominantemente trombocitopenia y hepatotoxicidad.

El estudio de fase III PHENIX ^36^ y el estudio PHOEBE ^35^ también han contribuido significativamente al conocimiento actual de estas terapias. El estudio PHENIX demostró una ventaja estadísticamente significativa en la SLP para la combinación de pirotinib y capecitabina en comparación con capecitabina más placebo. El estudio PHOEBE descubrió que la combinación de pirotinib y capecitabina era más eficaz en la mediana de la SLP (aproximadamente seis meses). Sin embargo, es importante señalar que estos estudios con pirotinib y capecitabina se realizaron exclusivamente en la población china, y los resultados deben ratificarse en otras poblaciones fuera de China. El último esquema con tendencia a ser superior en nuestro estudio en términos de SG, SLP y TRO es la combinación de T-DM1 y capecitabina. En el estudio de fase II TRAXHER247, el brazo de T-DM1 y capecitabina no demostró ser mejor que la monoterapia con T-DM1 en el criterio de valoración principal del ensayo clínico, ni tampoco en la SLP. Esta combinación tampoco ha sido aprobada por los organismos reguladores.

La fortaleza de nuestra revisión sistemática y NMA radica en la posibilidad de capturar y analizar toda la evidencia disponible, incluyendo los ECAs más recientes (con bajo riesgo de sesgo) sobre los diferentes esquemas de tratamiento en pacientes con cáncer de mama HER-2+ avanzado o locorregionalmente avanzado que progresan a trastuzumab y taxanos, así como su reproducibilidad. Por otro lado, permitió establecer un ranking terapéutico basado en la eficacia y seguridad comparativa entre las múltiples intervenciones disponibles. En resumen, T-DM1 presentó una SLP, una TRO y una tendencia a la SG inferiores a las de T-DXd, que dominó todos los esquemas estudiados en pacientes con cáncer de mama HER2+ avanzado o locorregionalmente avanzado en pacientes con progresión previa a trastuzumab y taxanos. Por otra parte, en el análisis de seguridad, T-DM1 se asoció a un perfil de toxicidad más favorable, con menores tasas de interrupción debida a EA relacionados con el fármaco y menos EA adversos de grado III/IV entre los regímenes de tratamiento más eficaces.

Las principales limitaciones de esta revisión sistemática incluyen la escasez de pruebas directas entre diferentes comparaciones de regímenes terapéuticos, lo que dio lugar a una red de pruebas limitada y a la imprecisión de las estimaciones, y también la presencia de heterogeneidad en varios resultados. Por otro lado, los datos de SG pueden estar muy influidos por la disponibilidad y el uso de líneas de tratamiento posteriores y las principales toxicidades de los tratamientos evaluados. Aunque las estimaciones centrales del análisis indican variaciones en la eficacia de los distintos tratamientos, la confianza general en estos resultados se ve atenuada por las incertidumbres subyacentes.

Una de las cuestiones clave es la inclusión de ensayos clínicos de varias fases, que intrínsecamente poseen diseños y objetivos diferentes. Esta diversidad en el diseño de los estudios implica la ausencia de muchas comparaciones directas entre tratamientos, lo que resta fuerza a los resultados globales y puede sesgar los resultados en una dirección desconocida. Además, a pesar de ser ECA, algunos de estos estudios pueden albergar sesgos que podrían influir en sus resultados, complicando aún más la interpretación del metaanálisis. Las características específicas de los pacientes y las líneas de tratamiento previas podrían influir en los resultados. Otra limitación importante es el ámbito temporal de la investigación. La búsqueda de estudios relevantes se realizó únicamente hasta el 5 de mayo de 2021. En consecuencia, cualquier novedad o estudio adicional publicado después de esta fecha no se refleja en el análisis, omitiendo potencialmente datos cruciales que podrían afectar a las conclusiones generales. Además, preocupa el cumplimiento de ciertos supuestos inherentes a los NMA, como la transitividad. La transitividad supone que los efectos de los tratamientos pueden compararse de forma fiable indirectamente a través de un comparador común en todos los estudios. Sin embargo, si este supuesto no se cumple en algunas comparaciones, podría dar lugar a conclusiones cuestionables sobre la eficacia relativa de los tratamientos.

En conclusión, aunque T-DM1 sigue siendo una piedra angular en el tratamiento del cáncer de mama HER-2+ avanzado o locorregionalmente avanzado, especialmente tras la progresión con trastuzumab y taxanos, ahora se ve desafiado por terapias más nuevas como T-DXd. T-DXd ha demostrado superioridad en SLP, TRO y una tendencia hacia una mejor SG. Sin embargo, en términos de seguridad, T-DM1 presenta un perfil más favorable, con menores tasas de interrupción debido a EA relacionados con el fármaco y menos EAG en comparación con los regímenes de tratamiento más eficaces. Esta evolución del panorama terapéutico subraya la necesidad de seguir investigando y adaptando las estrategias de tratamiento del cáncer de mama HER-2+ avanzado.
